# Motoneurone synchronization for intercostal and abdominal muscles: interneurone influences in two different species

**DOI:** 10.1007/s00221-020-05924-6

**Published:** 2020-10-26

**Authors:** J. D. Road, A. T. R. de Almeida, P. A. Kirkwood

**Affiliations:** 1 Division of Respiratory Medicine, Department of Medicine, University of British Columbia, Vancouver, BC V5Z1M9 Canada; 2 Sobell Department of Motor Neuroscience and Movement Disorders, UCL Institute of Neurology, Queen Square, London, WC1N 3BG UK; 3 Clinical and Movement Neurosciences, UCL Queen Square Institute of Neurology, Queen Square, London, WC1N 3BG UK

**Keywords:** Motoneuron synchronization, Thoracic motoneurons, Abdominal motoneurons, Spinal cord interneurons, Cross-correlation

## Abstract

The contribution of branched-axon monosynaptic inputs in the generation of short-term synchronization of motoneurones remains uncertain. Here, synchronization was measured for intercostal and abdominal motoneurones supplying the lower thorax and upper abdomen, mostly showing expiratory discharges. Synchronization in the anaesthetized cat, where the motoneurones receive a strong direct descending drive, is compared with that in anaesthetized or decerebrate rats, where the direct descending drive is much weaker. In the cat, some examples could be explained by branched-axon monosynaptic inputs, but many others could not, by virtue of peaks in cross-correlation histograms whose widths (relatively wide) and timing indicated common inputs with more complex linkages, e.g., disynaptic excitatory. In contrast, in the rat, correlations for pairs of internal intercostal nerves were dominated by very narrow peaks, indicative of branched-axon monosynaptic inputs. However, the presence of activity in both inspiration and expiration in many of the nerves allowed additional synchronization measurements between internal and external intercostal nerves. Time courses of synchronization for these often consisted of combinations of peaks and troughs, which have never been previously described for motoneurone synchronization and which we interpret as indicating combinations of inputs, excitation of one group of motoneurones being common with either excitation or inhibition of the other. Significant species differences in the circuits controlling the motoneurones are indicated, but in both cases, the roles of spinal interneurones are emphasised. The results demonstrate the potential of motoneurone synchronization for investigating inhibition and have important general implications for the interpretation of neural connectivity measurements by cross-correlation.

## Introduction

It is widely accepted that two motoneurones engaged in a common task will be more likely to fire within a few ms of each other than chance would predict, i.e., their discharges will be, to an extent, synchronized. This is particularly the case for motoneurones innervating the same muscle, but is also true for close agonists and even sometimes for anatomical antagonists. In one of the first descriptions of the phenomenon, Sears and Stagg (1976), working on inspiratory intercostal discharges in the cat, wrote “If such synchronization does occur and the circumstances for reliably detecting it could be defined, then one could take advantage of this physiological property to infer from the output of motoneurones certain properties of their presynaptic inputs”. The clear implication, as pointed out by Sears and Stagg, was that this gave some insights into the properties of human motoneurone inputs that would be otherwise inaccessible.

In the years since, this logic has gained considerable traction and many studies in human subjects have followed. The early analyses, in the time-domain (Milner Brown et al. 1973; Datta and Stephens 1990), were rapidly supplemented or replaced by frequency-domain approaches (Farmer et al. 1993), where coherence analyses replaced cross-correlation histograms. Whereas the time-domain approach concentrated on “short-time synchronization” (STS), regarded by Sears and Stagg (1976) and by Milner Brown et al. (1973) as an inevitable consequence of the known branching of presynaptic axons to innervate a high proportion of the motoneurones of a given motor nucleus, the frequency-domain approach has revealed other features of motor drives, such as rhythmicities, that likely originate in higher centres. Thus, the interest of these studies has been in rather general properties of motor control, together with the possible functional significance of the synchronization itself.

In contrast, STS, interpreted in terms of the immediate or near-immediate inputs to motoneurones, has received less attention. Nevertheless, it has continued to be a feature of motoneurone synchronization studies. In particular, when the correlation peaks have been strong and narrow, they have been taken as indicating strong corticospinal inputs, (for refs: Farmer et al. 1997; Semmler et al. 2006), although additional sources of inputs for STS have also been thought necessary (Keen et al. 2012) and STS has been shown in spinal preparations (Connell et al. 1986; Nielsen et al. 2005). The evidence supporting the link to the corticospinal input, though plentiful, is all indirect, but is attractive because of the functional significance, in primates, of the direct corticomotoneuronal connections (Lemon 2008). A possible analogy may be found in the cat intercostal motoneurones originally studied by Sears and Stagg (1976). This analogy was first apparent in the study of Datta et al. (1991), where the presence of STS in normal subjects but the appearance of “broad-peak” synchronization in patients with CNS lesions was related to the similar contrast reported by Kirkwood et al. (1982b, 1984) between the STS associated with descending respiratory drive and broad-peak synchronization associated with likely spinal interneurone inputs. The analogy has been complicated somewhat by Vaughan and Kirkwood (1997) making a distinction between the shortest duration forms of STS, which they interpreted as resulting from branched-axon monosynaptic connections, and those with rather longer durations, taken as representing common disynaptic connections. The shortest duration form comprised histogram peaks with half-widths (durations at half amplitude) up to 2.1 ms, and was derived from the discharges of phrenic motoneurones and/or of those innervating parasternal muscle, which both receive strong monosynaptic bulbospinal inputs. The rather longer duration peaks were derived from the discharges of external intercostal motoneurones, for which such connections are much weaker. Nevertheless, in either case, human corticospinal or cat inspiratory drive, the association has been made, correctly or not, between the “purest” (branched-axon monosynaptic) form of common input and activity in long descending axons of supraspinal origin.

Another set of long descending axons giving monosynaptic connections in the cat are the bulbospinal connections to expiratory motoneurones (internal intercostal and abdominal). These connections are present in many segments, extending at least from T7 to L1 (Cohen et al. 1985; Kirkwood 1995; Saywell et al. 2007; Road et al. 2013) and are strong, as indicated by spike-triggered averaging or cross-correlation, comparable in strength to the inspiratory connections to the phrenic motoneurones. In the rat, equivalent connections are present although much weaker, but they are accompanied by disynaptic connections from the same bulbospinal neurones to external intercostal motoneurons (de Almeida and Kirkwood 2013). In the present study, we have therefore investigated the motoneurone synchronization present for the nerve discharges of the expiratory motoneurones in the two species, to ask whether the association between a strong monosynaptic input from long descending fibres and the the branched-axon monosynaptic form of synchronization may be retained in these instances. In addition, the discharges in the rat involve temporally overlapping activation of internal and external intercostal muscles (most often regarded as antagonists) along with, in at least some motoneurones, simultaneous excitation and inhibition (de Almeida et al 2010; de Almeida and Kirkwood 2010). Thus, a second aim of the study was to investigate how these more diverse patterns of activation may be represented in synchronization measurements.

The results, which included relatively strong synchronization of the branched-axon monosynaptic form between some classes of rat motoneurones, have not supported the proposed association, but the measurements in the rat have revealed a variety of never previously reported time courses for STS, including presumed inhibitory effects. For both cat and rat, the measurements emphasise, differently for the two species, the contributions from interneurones in the transmission of the respiratory drive to the motoneurones.

Preliminary results have been published (Kirkwood and Road 1994).

## Methods

### Animals

Experiments were conducted as approved by the Ethical Review Process of the Institute of Neurology, in accordance with UK legislation [Animals (Scientific Procedures) Act 1986] under Project and Personal Licences issued by the UK Home Office. The data come from 12 adult cats (6 male), weights 2.1–3.85 kg, from the series already described by Road et al. (2013), together with 11 adult female Sprague–Dawley rats (Harlan, UK), weights 180–254 g, from the series already described by de Almeida and Kirkwood (2013).

### Cat preparations

Animals were anaesthetized with sodium pentobarbitone (initial dose 37.5 mg kg^−1^ I.P., then I.V. as required), maintained under neuromuscular blockade with gallamine triethiodide (subsequent to surgery, repeated doses, 24 mg I.V., as required) and artificially ventilated via a tracheal cannula with O_2_-enriched air, so as to bring the end-tidal CO_2_ fraction initially to about 4%. CO_2_ was then added to the gas mixture to raise the end-tidal level to a value sufficient to give a brisk respiratory discharge in the mid-thoracic intercostal nerves (typically 6–7%). A low stroke volume and a high pump rate (53 min^−1^) were employed, so that events related to the central respiratory drive (Sears 1964b) could be distinguished from those due to movement-related afferent input. Venous and arterial cannulae were inserted.

**Fig. 1:**
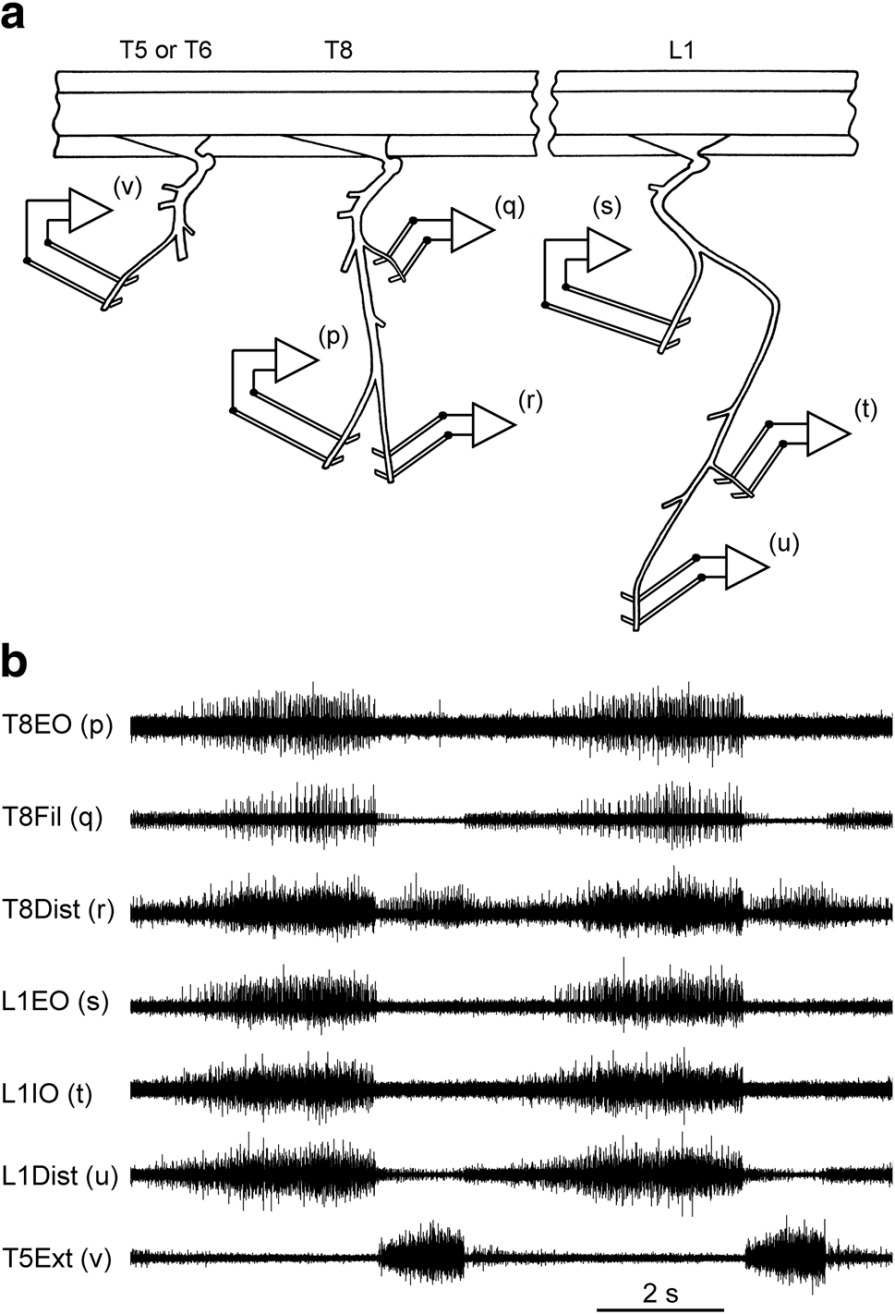
Raw data from a cat experiment. **a** Schematic of the recording set-up. The efferent discharges in the 6 nerves (p)–(u) were used for measurements of motoneurone synchronization by cross-correlation. The 7th nerve (v), the external intercostal nerve at T5 or T6, was used as a marker of central inspiration. **b** A typical recording from these 7 nerves. (p)–(r) Three branches of the internal intercostal nerve at T8: (p) the lateral branch, innervating external abdominal oblique; (r) the distal remainder; (q) the first filament (Sears 1964a). (s)–(u) Three branches of L1 ventral ramus: (s) the lateral branch, innervating external abdominal oblique; (t) a branch innervating internal abdominal oblique; (u) a distal remainder

We aimed to use a (surgically adequate) level of anaesthesia in the range light to moderately deep, as described by Kirkwood et al. (1982b). Before neuromuscular blockade, a weak withdrawal reflex was elicited by noxious pinch applied to the forepaw, but not to the hind paw. Pinch-evoked changes in blood pressure (measured via a femoral arterial cannula), were absent or were small and of short duration. During neuromuscular blockade, anaesthesia was assessed by continuous observations of the patterns of the respiratory discharges in the intercostal nerves and blood pressure together with responses, if any, of both of these to a noxious pinch of the forepaw. Only minimal, transient responses (similar to those before neuromuscular blockade) were allowed before supplements (5 mg kg^−1^) of pentobarbitone were administered. The responses to a noxious pinch always provided the formal criteria, but, in practice, the respiratory pattern, indicated by an external intercostal nerve discharge that was continuously monitored on a loudspeaker from the induction of neuromuscular blockade, always gave a premonitory indication of the need for an anaesthetic supplement. Any increase from the usual slow respiratory rate typical of barbiturate anaesthesia (e.g., Fig. 1) led to such a test being carried out. The animal was supported, prone, by vertebral clamps, a clamp on the iliac crest and a plate screwed to the skull. Rectal temperature was maintained between 37 and 38 °C by a thermostatically controlled heating blanket. The bladder was emptied by manual compression at intervals. Systolic blood pressures were above 80 mmHg throughout, maintained in a few animals by occasional infusions of 5% dextran in saline. At the end of the experiment, the animals were killed with an overdose of anaesthetic.

Nerves on the left side, which innervate muscles with an expiratory function, were prepared for recording efferent discharges from their cut central ends via platinum wire electrodes, as illustrated in Fig. 1a. At T8: (p) the lateral branch of the internal intercostal nerve, which innervates external abdominal oblique (T8EO); (q) one of the filaments of the internal intercostal nerve, which are the naturally occurring branches that leave the nerve at intervals to innervate the internal intercostal muscle layer (Sears 1964a), usually the most proximal one (T8Fil); (r) the distal remainder of the internal intercostal nerve (T8Dist), which innervates the more distal part of the internal and parasternal intercostal muscles, transversus abdominis and rectus abdominis. At L1, branches of the ventral ramus: (s), the major branch which innervates external abdominal oblique (L1EO); (t) one of the more distal branches innervating internal abdominal oblique (L1IO); (u) a distal remainder, which innervated transversus abdominis, but probably also innervated more of internal oblique muscle, and rectus abdominis (L1Dist). Finally, (v) the external intercostal nerve of T5 (T5Ext) or T6 (T6Ext) was also prepared, whose efferent discharges were used to define the timing of central inspiration. Road et al. (2013) and Meehan et al. (2004) should be consulted for fuller descriptions and references. In one of the animals, an additional internal intercostal nerve filament was prepared from T9 (T9Fil) and in another, the lateral nerve at T9 (T9EO). In two further animals, no lumbar nerves were dissected, but the three nerves (p–r) from both T8 and T9 were prepared, and in the final animal, the two nerves (p, q) were prepared from both T8 and T9. The thoracic nerves were recorded under paraffin oil, in a pool constructed from skin flaps. The lumbar nerves were recorded under petroleum jelly, most often with a piece of thin plastic film separating the electrodes from underlying muscle.

### Rat preparations

A range of different anaesthetic regimens were used, which are listed in Table 1, together with the numbers of animals used under each regimen. The different regimens represent successive trials in establishing preparations suitable for cross-correlation measurements between the discharges of expiratory bulbospinal neurones (EBSNs) and motoneurones (de Almeida and Kirkwood 2013), as described by de Almeida et al. (2010). Atropine sulphate (60 µg, I.M., Hameln Pharmaceuticals Ltd, UK) was administered to minimize airway fluid secretion. Rectal temperature was maintained at 37–38 °C with a thermostat-controlled heating blanket (Harvard). The jugular vein was cannulated for the administration of anaesthetic supplements and fluids, and the trachea for mechanical ventilation. The right carotid artery was cannulated for the measurement of blood pressure and bilateral vagotomies were performed. In the decerebrate experiments, the left carotid artery was also prepared, with a loose tie around it. Sodium lactate solution (Hartmann’s solution, Baxter Healthcare Ltd.) was administered periodically (up to 1 ml h^−1^) and a plasma substitute (Gelofusin, Braun Medical Ltd.) was infused as required (both I.V.), to stabilize the animal’s fluid balance and to maintain blood pressure within physiological limits. A mean arterial pressure above 80 mm Hg was maintained for nearly all of the recordings, though occasionally, towards the end of an experiment, a decline to 60 mm Hg was accepted, but only if the pattern of respiratory discharges remained stable (de Almeida et al. 2010).

**Table 1:** Anaesthetic regimens employed for rat experiments

Anaesthetic and dose	No of experiments
Halothane only Induction 5%, maintenance 1–2% for surgery and 0.4–1% for recording	1
Fentanyl and ketamine, then α-chloralose Induction, fentanyl, 2 mg kg^−1^, plus ketamine 50 mg kg^−1^, I.P., maintained with ketamine and fentanyl I.P, then I.V. as required. For recordings, α-chloralose (20 mg kg^−1^, I.V.)	1
Halothane then α-chloralose Halothane: Induction 5%, maintenance 1–2% during surgery, titrated to α-chloralose (30 – 80 mg kg^−1^, I.V.) for recording	2
Ketamine/xylazine then decerebration Induction, I.P., 100 mg kg^−1^, 10 mg kg^−1^ respectively, then supplements of ketamine/xylazine (same proportions), I.V. as required, until decerebration	4
Halothane then decerebration Induction 5%, maintenance 1–2% during surgery. Lowered to 0.5% at start of decerebration; turned off once decerebration completed	3

Suppliers: ketamine, Vetalar, Pfizer, UK; xylazine, Rompun, Bayer plc, UK; urethane, Sigma; halothane; Merial Animal Health Ltd, UK; Fentanyl, Sublimaze, Janssen–Cilag, UK; α-chloralose; α-Chloralose-HBC complex, Sigma (doses cited above are the equivalent doses of α-chloralose; actual doses of the complex were 10 × higher)

For the recordings, neuromuscular blockade was used (pancuronium bromide; 0.3 mg h^−1^ I.V*.,* Hospira UK Ltd). Before neuromuscular blockade, anaesthesia was maintained at a level where the animal showed no more than a minimal withdrawal reflex to a noxious paw pinch. After neuromuscular blockade, depth of anaesthesia was assessed from the recordings of blood pressure and the respiratory discharges. Only minimal transient changes of blood pressure, heart rate, or respiratory pattern following a similar pinch were allowed before anaesthetic supplements were administered. Particular care was taken to monitor anaesthetic levels with the use of ketamine/xylazine, where frequent supplements were needed (de Almeida et al. 2010). Animals were supported, prone, by vertebral clamps usually at T6 and T12 and by a strong ligature tied into the lumbar fascia. The head was supported in anaesthetized animals by a plate screwed to the skull and in decerebrate preparations by a custom-made clamp with bilateral spikes screwed into the frontal and premaxillary bones. Artificial ventilation was carried out using O_2_-enriched air at a rate of 100 min^−1^. Expired CO_2_ was monitored at the trachea, the stroke volume adjusted to bring the end-tidal CO_2_ fraction to about 4%, and then CO_2_ was added to the gas mixture to increase the end-tidal CO_2_ level to a value adequate to give brisk respiratory discharges in the intercostal nerves, usually around 7–8%.

External and internal intercostal nerves on the left side (see below) were dissected and their cut central ends were mounted for recording on pairs of platinum wire electrodes in a paraffin oil pool constructed from skin flaps. These nerves are formally described by Smith and Hollyday (1983) and de Almeida et al. (2010), but the anatomy is very similar to that in the cat (Fig. 1).

For decerebration, the left carotid artery was tied. A wide craniotomy was made, with ligation of the sagittal sinus. Animals were artificially ventilated and all brain tissues rostral to the superior colliculus was removed by use of a blunt spatula and aspiration. To minimise further bleeding, the exposed brainstem was gently covered with Spongostan (Johnson & Johnson Medical, UK) soaked in saline.

At the end of the experiment, the animal was killed with an anaesthetic overdose, the particular substance usually being that used in the earlier part of the experiment (but for decerebrates following halothane, pentobarbital sodium, Lethobarb, Fort Dodge Animal Health, UK, was used). Following anaesthetic administration, zero blood pressure was confirmed for 5 min.

### Procedures and analysis

For both the cat and rat, as previously described (Road et al. 2013; de Almeida and Kirkwood 2013), one of the original aims of the experiments was to measure connections between EBSNs and motoneurones, using cross-correlation between their discharges. Thus, all of the rat data and nearly all of the cat data come from simultaneous recordings of nerve discharges, via conventional band-pass recordings (300 Hz to 3 kHz), together with the activity of an EBSN, stored on magnetic tape.

There are important differences in the patterns of discharges in the intercostal nerves between cat and rat (de Almeida et al. 2010). Thus, although the basic analyses were the same for the two experimental series, there were differences in detail. The first difference was in the nerves recorded. For the rat, in place of the 7 nerves illustrated in Fig. 1b, these were most often 4 nerves: (a) the whole internal intercostal nerve (Int) in two adjacent intercostal spaces, usually T9 and T10, recorded distal to the most proximal filament; (b) the whole external intercostal nerve (Ext) in one of those segments and in T6. In one animal, the first filament of the internal intercostal nerve (Fil) was included in place of one of the Int recordings. More details can be found in de Almeida et al. (2010). Second, there were matters related to spike selection. For the cat data, spikes were selected, by amplitude, to be those of α motoneurones, as in Kirkwood (1995) and in Road et al. (2013). For the rat data, we do not have suitable criteria for discriminating between α and γ spikes, though it is almost certain that most of our recordings included both categories, as discussed by de Almeida et al. (2010). Nevertheless, we chose to select spikes that were larger than a certain amplitude (generally those that were phasically active with respiration), typically with a level around 50% of the amplitudes of largest ones, as being likely to be similar to those selected as being α in the cat.

**Fig. 2:**
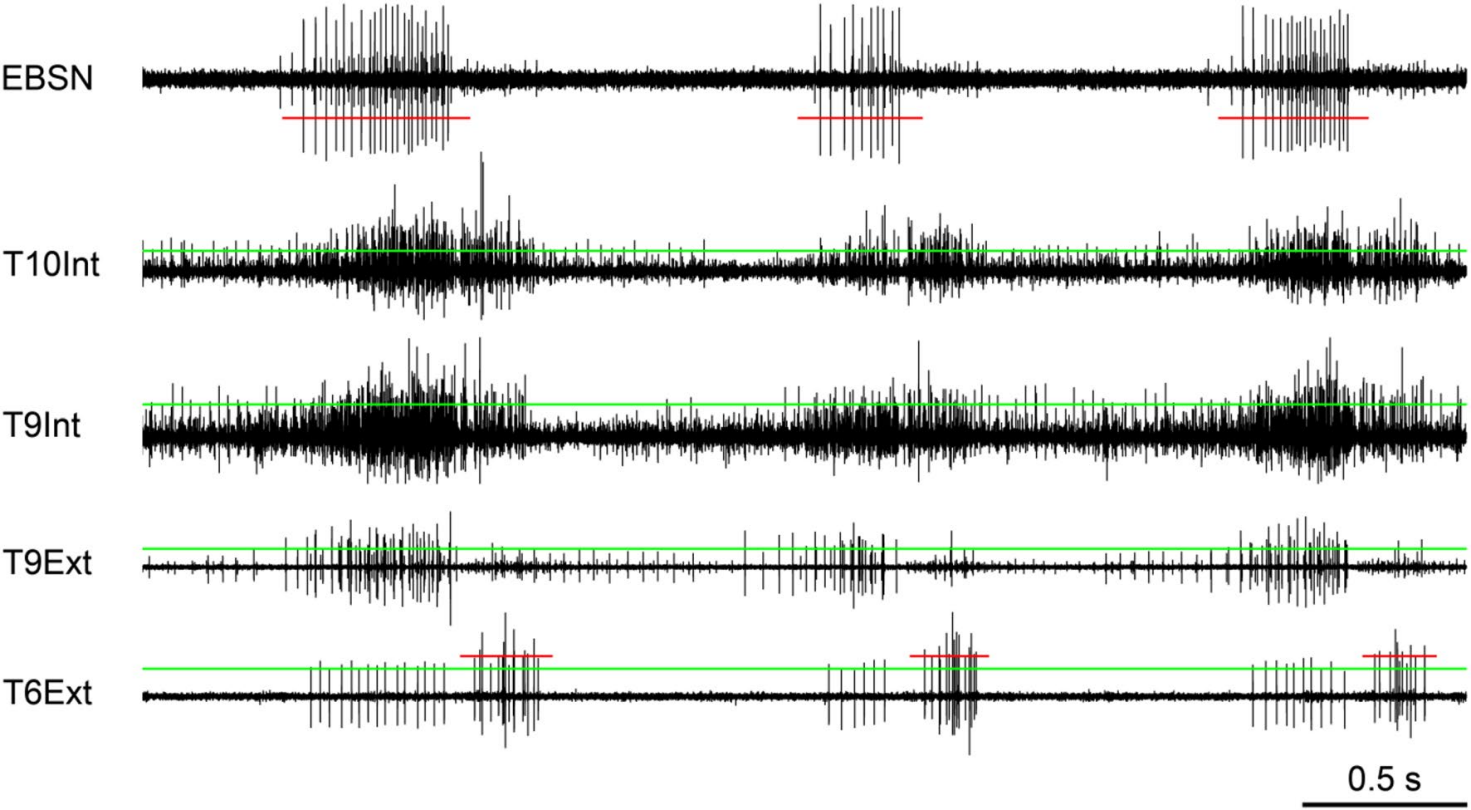
Raw data and spike selection from a rat experiment. The four lowest traces show efferent discharges recorded from the indicated nerves. The continuous (green) horizontal lines show the thresholds (positive-going) for registering spikes as events. The large spikes in the top trace (EBSN recording) were used in this recording for identifying periods designated as expiration, using the threshold (negative-going) indicated by the interrupted (red) line. The large spikes in the lowest trace, with their threshold (positive going) indicated by the interrupted (red) line, were used for identifying periods designated as inspiration. See text for more details. α-chloralose anaesthesia

Second, as previously described (de Almeida et al. 2010), in recordings from either or both of internal and external intercostal nerves, phasic bursts of discharges may be present in both inspiration and expiration, as shown in Fig. 2. This example is from a chloralose-anaesthetized animal, but, despite the variation from preparation to preparation in terms of the balance of expiratory and inspiratory activation in any particular nerve, for the animals used in these analyses, there were no obvious differences in the patterns between the anaesthetized animals and the decerebrates. The animals used here were a sub-group of those described by de Almeida and Kirkwood (2010), corresponding closely to those also reported by de Almeida et al. (2013), i.e., 3 of the 4 anaesthetized animals and all 7 of the decerebrates in that study, together with one additional anaesthetized and one additional decerebrate.

Data were subsequently re-acquired for computer analysis. For nine of the cat experiments, the acquisition was made via a purpose-built interface (Davies et al. 1983) which included window discriminators in each of eight channels, so that events corresponding to presumed α motoneurone spikes from each nerve recording were selected, as in Road et al. (2013). For the remaining 3 cat experiments and the rat experiments, the nerve recordings were acquired as analogue signals (sampled at about 10 kHz) via a 1401 interface and Spike2 software (CED, Cambridge). Events corresponding to the times of presumed α motoneurone spikes in the cats or the larger amplitude spikes in the rats were subsequently derived in Spike2. Cross-correlation histograms were constructed between the event times in the different channels, using bin-widths of about 200 µs (192–250 µs), choosing values as required to avoid aliasing, which could sometimes occur as interactions between the sampling interval of the analogue signal and the bin-width.

To simplify interpretation of the results in the rats, it was preferable to measure histograms in only one phase of respiration. Because of the usual occurrence of spikes in both phases of respiration in the recordings from this species, there was not a particular channel that could always be used as a marker of one phase or another, so different criteria were used in the different recordings. In 6 instances, the occurrence of large spikes in the T6Ext recording could be used to gate the selection of events for correlation to occur only in inspiration, the spikes in expiration often being smaller. Times outside of these periods were taken as expiration. However, in the example of Fig. 2, as was the case in some other recordings, the discharges of the large spikes could be quite sparse, sometimes starting quite late in inspiration, so this procedure was not reliable for defining expiration. However, in this instance, the EBSN recording was very consistent, firing only in the second half of expiration, and the times of its spikes were used as a gate signal instead. This gave non-overlapping periods for inspiration (via the large spikes in T6Ext) and expiration (via the EBSN spikes). Some spikes were missed in early inspiration and in the first half of expiration, but this conservative approach was preferred to the ambiguity arising from the possible overlap. The first half of expiration was, in any case, a time of low motoneurone activity. This combination was used in one other instance, and the EBSN discharge on its own in two others, defining inspiration in these two instances as the time outside the period of EBSN discharge. Finally, in two further instances, an additional channel of recording was available, from the T8Ext on the right side (not otherwise analyzed), which showed large spikes throughout inspiration, so was used to define that period. When a gating signal was used for acceptance (e.g., inspiratory spikes defining inspiration), only a short period around each spike (e.g., ± 10 or 20 ms) was employed for gating, but when used for rejection (e.g., absence of inspiratory spikes defining expiration), longer periods were employed, different in each instance, so that the gating period for rejection (e.g., inspiration) always equalled or exceeded the range of actual inspiratory times. This again led to some loss of spikes to be correlated, but was also preferred as a conservative approach.

The strength of the synchronization for the cat data from T8/L1 nerve pairs could be relatively weak. To obtain a sufficient signal-to-noise ratio, long virtual epochs of data were therefore constructed by adding together, where available, the histograms from several original recording runs, each corresponding to the recording of one EBSN. Thus, the lengths of these virtual epochs (one for each animal) ranged from 1750 to 24,282 s (median 7396 s). For the rat data, such long runs were not needed and usually a single run (or two sequential runs acquired continuously), again one for each animal, was used (720–5453 s, median 2209 s).

Peaks or troughs in the cross-correlation histograms were accepted as significant if a single bin differed from *m* by more than 3.29√*m* (Sears and Stagg 1976), where *m* was the baseline count, as in Davies et al. (1985a) and Kirkwood (1995). Single bin counts outside *m* ± 3.29√*m* (which will sometimes occur by chance) were ignored if they fell outside the range of expected latencies (± 10 ms). The strength of a peak was assessed by *k*, the ratio of the highest count within a peak to *m* (Sears and Stagg 1976; Davies et al. 1985a, b; Kirkwood 1995). The sensitivity of detecting a peak with a given value of *k* depends on *m*, and, therefore, on the length of the recording and the discharge rates of the EBSN and of the efferent population, all of which were highly variable between the different nerves and between different runs. To give some meaning to an absence of a peak, only histograms with *m* ≥ 121 were considered as tested, as in Kirkwood (1995). A baseline count of 121 allows the detection of peaks with *k* ≥ 1.3. For the cat data, the histograms were smoothed with a 5-point triangular filter, to make then comparable to those of Vaughan and Kirkwood (1997). Smoothing of the histograms was not employed for the rat data, because many of the peaks apparent in the rat data were sharper, or included faster-rising or falling components than in the cat. These would have been significantly attenuated by smoothing.

In the descriptions of cross-correlation histograms that follow, the first-mentioned nerve always provided the reference events, e.g., T8Int/L1IO refers to a histogram where the times of L1IO spikes were measured relative to those of T8Int.

Mean values are quoted as ± S.D. In statistical tests, *P* < 0.05 was taken as significant.

## Results

### Cat experiments

**Fig. 3:**
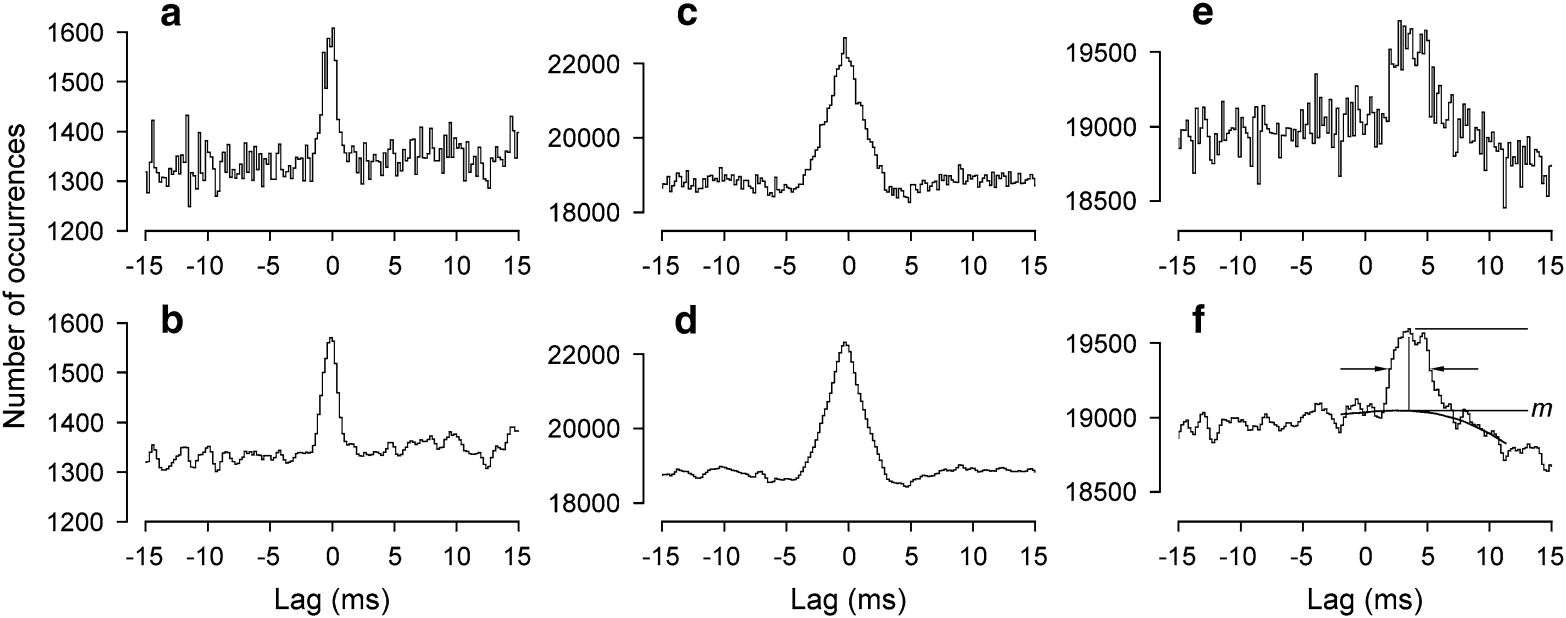
Examples of cross-correlation histogram peaks from the cat experiments with a variety of durations and timings. **a**, **c**, **e** Raw histograms. **b**, **d**, **f** Same histograms after smoothing (5-point triangular filter). **a**, **b** T9EO/T9Fil. **c**, **d** L1IO/L1Dist. **e**, **f** T8Dist/L1IO. The peak in **f** is annotated to show the parameters measured: the baseline, *m*; the amplitude of the peak, usually expressed as *k*, the peak count (upper horizontal line) divided by *m*; the half-width (time between the arrows); the lag to the peak (lag of the vertical line)

**Fig. 4:**
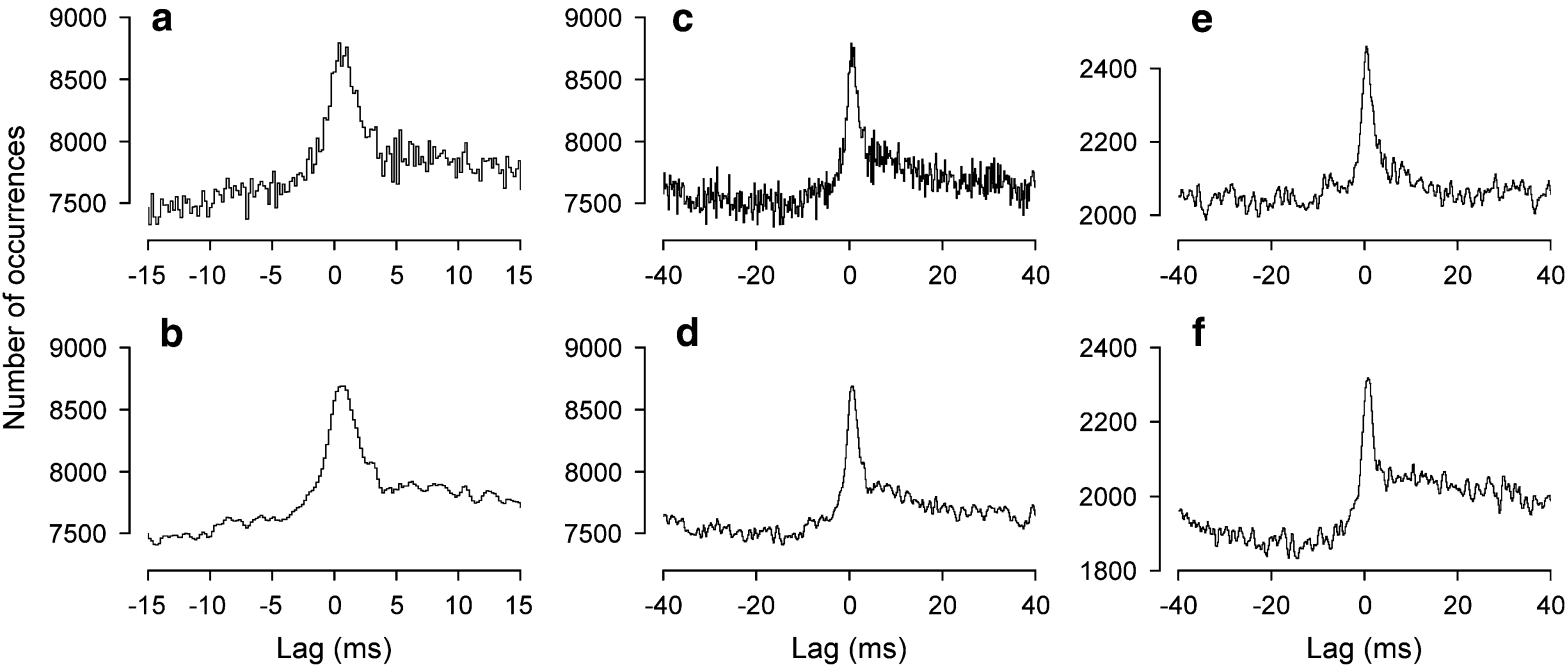
Distinguishing between short-term synchronization (STS) and broad-peak synchronization. Cross-correlation histograms from one pair of nerves (T8Fil/T8Dist) in one cat experiment. **a**, **c** The histogram derived from data during the whole experiment, shown at two different lag scales. **b**, **d** The same two plots after smoothing. **e**, **f** Smoothed histograms derived from data early (**e**) and late (**f**) in the experiment. Note the narrow peak (STS) is largely constant, but the underlying broad peak varies

**Fig. 5:**
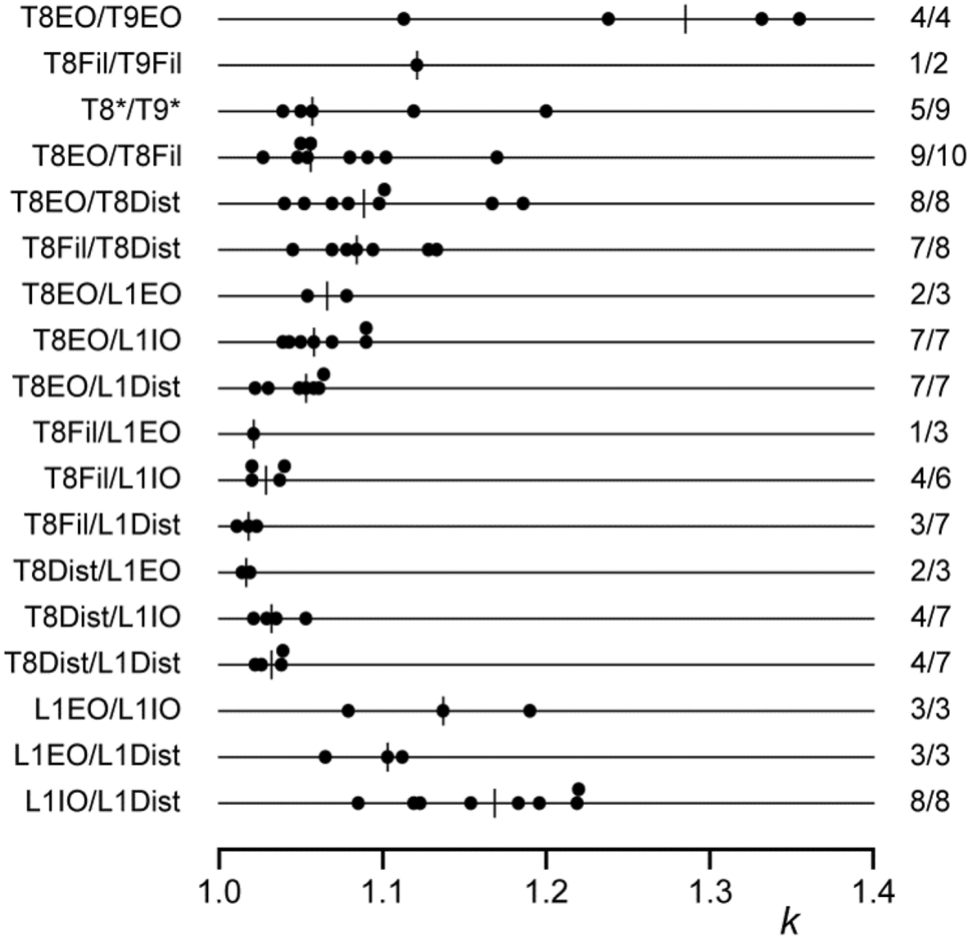
Amplitudes and occurrence of histogram peaks for the cat data. Values of *k* are plotted for each of the nerve pair categories, as indicated. T8*/T9* (third line) includes two examples of T8EO/T9Fil plus one each of T8Fil/T9Lat, T8EO/T9Dist, and T8Dist/T9Lat. The vertical bars indicate the median for the individual values shown by the points on each line. The right-hand column shows the proportion of experiments yielding a histogram with a significant peak, compared to the total tested for that category. The three ipsi-segmental thoracic nerve pair categories (lines 4–6) include four examples that were actually from T9

The analyses here involved the expiratory discharges of the larger amplitude, presumed α, spikes, phasically active with respiration. Only the T8Dist or T9Dist branches showed an inspiratory burst (Fig. 1b), and correlations were never performed between these two. Most of the nerve pairs analyzed gave cross-correlation histograms with narrow central (near zero lag) peaks, i.e., they showed STS. Three examples, illustrating some of the variation seen in the time courses of the peaks, are included in Fig. 3, which also illustrates the measurements of the baseline count, *m*, the amplitude of the peak, *k* (ratio of peak value to *m*), and the half-width (duration of the peak at half amplitude). These were all measured from the smoothed versions of the histograms, but note that the statistical test for the presence of a peak (see “Methods” section) was based on the bin counts before smoothing. For a sloping or curved baseline, as illustrated in (f), *m* was measured (at the same lag as the peak) from an interpolated baseline fitted by eye to the region of the histogram encompassing the peak, as previously (Davies et al. 1985a; b; Kirkwood 1995; Vaughan and Kirkwood 1997). A further example (Fig. 4) illustrates the usual origin of sloping or curved baseline, namely the additional presence of a longer duration peak (extending in this example from a lag of about − 15 to + 20 ms, or beyond), upon which the narrow peak was superimposed. Such a feature was interpreted as being equivalent to similar features (“broad peaks”) seen in synchronization measurements for external intercostal motoneurone discharges (Kirkwood et al. 1982b). As in that previous study, broad peaks could sometimes be seen to vary independently of the narrow peaks. Figure 4 includes examples of histograms obtained from the same nerves relatively early in an experiment (e) and late (f). By analogy with examples analyzed by Kirkwood et al. (1982b), the variation in the broad peaks most likely represents a state dependence, such as a variation in the depth on anaesthesia during the different recording runs. Only a minority of nerve pairs showed obvious broad peaks. Figure 4 is one of the strongest examples. They tended to be larger, relative to the narrow peaks, for the T8/L1 nerve combinations (e.g., Fig. 3e, f), probably because the narrow peaks were generally weaker for these pairs than for the ipsi-segmental pairs (Fig. 5).One hundred and five nerve pairs were counted as being tested for the presence of narrow peaks (i.e., with *m* ≥ 121), of which 82 yielded histograms where those peaks were significant (see “Methods” section). Half-widths varied between 1.3 and 4.6 ms and maximum values were at lags between − 0.7 to 5 ms. Forty-one of the pairs involved two thoracic nerves (34 with peaks), 14 involved two lumbar nerves (all with peaks) and the remaining 50 (34 with peaks) involved one T8 nerve and one L1 nerve.

#### Amplitudes of histogram peaks

The amplitudes of the histogram peaks ranged from *k* = 1.011 to *k* = 1.355, a similar range to that for the external intercostal nerves in Kirkwood et al. (1982a, b). Figure 5 indicates how those values were distributed between the different populations of nerve pairs, grouped according to the segments and nerves involved. The largest values were seen, not within a segment, but for the EO nerve in adjacent segments, T8EO/T9EO, with also the single value for T8Fil/T9Fil (the only other pair of homologous nerve branches in adjacent segments) being in the same range (upper two lines in Fig. 5). The values from the three L1 pairs (lowest 3 lines) were also large, with the median for L1IO/L1Dist being the largest. Next come a miscellaneous group of pairs of different nerves from adjacent segments (T8*/T9*), together with the pairs with both nerves in T8 or in T9, all of these having rather similar distributions (3rd to 6th lines). Finally, the remaining group of pairs involved one nerve from T8 and one from L1. A notable difference was seen here, where the values from the three pairs involving T8EO with each of the L1 nerves were generally larger than those for the other six pairs.

The point of interest with regard to these differences is that it appears as if the particular muscle innervated has importance in determining *k*. Thus, the median value for T8EO/T9EO, involving two nerves innervating the same muscle, was much larger than for any of the ipsi-segmental T8 or T9 combinations, which involved heterogenous nerve pairs. Similarly, L1Dist probably includes some distal branches innervating IO muscle, so it is then appropriate that L1IO/L1Dist gave a larger median value than the other two L1 pairs for which there was not a muscle in common. Finally, it is striking that the 3 T8EO/L1 nerve pairs gave generally larger values than the other 6 T8/L1 pairs, the T8EO nerve being the one T8 nerve that innervates only an abdominal muscle. Several of the other minor differences in Fig. 5 might be interpreted along similar lines, but the differences and the numbers of observations are small. The above three comparisons were confirmed statistically (Mann–Whitney, two-tailed: *P* < 0.00006 either for the 4 T8EO/T9EO pairs alone, or also including the one T8Fil/T9Fil, compared with the 24 ipsi-segmental pairs; *P* < 0.02 for the 8 L1IO/L1Dist pairs compared with the other 6 L1 pairs; *P* < 0.00006 for the 16 T8EO/L1 pairs compared with the remaining 18 T8/L1 pairs).

Note also that the proportion of pairs showing a significant peak (right-hand column in Fig. 5) generally co-varied with the amplitude measurements, further exaggerating the differences above, in particular between the T8EO/L1 nerve pairs and the other T8/L1 nerve pairs.

#### Time courses of histogram peaks

**Fig. 6:**
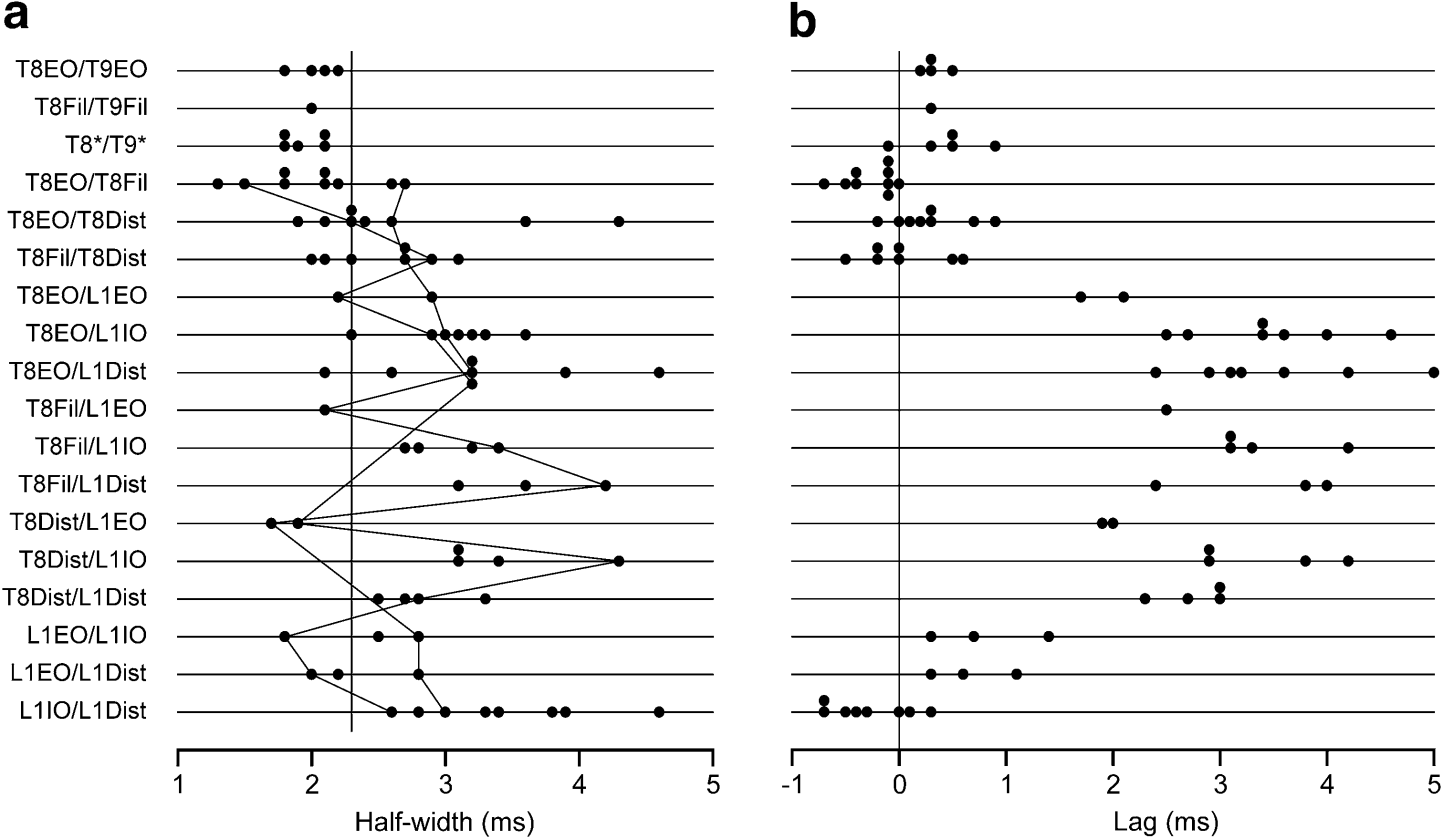
More properties of the same histogram peaks summarized in Fig. 5. **a** values of half-width (same format as Fig. 5). Lines join points from two particular experiments, to show that the low values for T8/L1EO categories do not represent generally low values for those individual preparations. Vertical line indicates half-width value of 2.3 ms. **b** values for the lags to the peaks

The interest in the time courses is that, following Vaughan and Kirkwood (1997), we wish to interpret peaks with the shortest durations as arising from mostly branched-axon monosynaptic common inputs to the two groups of motoneurones, half-widths > 2.1 ms then being regarded as including components of disynaptic, or more complicated common input circuits. Here, we can use a slightly modified criterion, since some of the thoracic nerves used, the EO and Dist branches involved longer conduction distances from the spinal cord than those used by Vaughan and Kirkwood (1997). Thus, for the branched-axon interpretation for thoracic ipsi-segmental or adjacent segmental pairs, we have increased this borderline to 2.3 ms, to allow for the additional temporal dispersion in these nerves. Measured half-widths are summarized in Fig. 6a.

For the thoracic nerve pairs, all of the adjacent segment pairs and many of the ipsi-segmental pairs had half-widths at or below 2.3 ms, but for a substantial proportion of the ipsi-segmental ones (10/24), the half-widths were longer (Fig. 6a, lines 4–6). Overall, the distribution of half-widths for thoracic nerve pairs (range 1.3–4.3 ms) was very similar to that for the pairs of adjacent segment external intercostal (inspiratory) nerves in Vaughan and Kirkwood (1997), which were interpreted as arising largely via common disynaptic inputs.

For the lumbar nerve pairs, the half-widths were generally longer, although for the 2 pairs involving L1EO, not excessively so. The L1EO nerves were short, comparable to the proximally located recordings of Vaughan and Kirkwood (1979) or the T8Fil recordings here, but the L1IO or L1Dist nerves were considerably longer. From the data of Road et al. (2013), the temporal dispersion in these nerves for presumed alpha spikes ranged from 0.35 to 0.82 ms, so it would not be unreasonable to expect, from this mechanism, an increase in half-width of around 0.4 ms for the L1EO/L1IO and L1EO/L1Dist peaks, i.e., a limit for a monosynaptic branched-axon explanation of 2.5 ms. Four out of six of the examples of these pairs correspond to this. For L1IO/L1Dist, even longer half-widths might be expected, up to around 2.9 ms. The lowest two values of half-width for this group (2.6 and 2.8 ms) are within this, but the others, 3.0–4.6 ms are longer.

A wide range of values for half-width was also seen for the T8/L1 nerve pairs. However, again note that the half-widths of peaks involving L1EO were generally less than those from the rest. Indeed, 4/5 of them were less than 2.3 ms, as appropriate to the monosynaptic branched-axon model, even without having to invoke the additional temporal dispersion which was likely to have arisen from varied conduction velocities in the presynaptic axons over the six segments separating these nerve pairs. All but one of the other T8/L1 examples showed a half-width greater than 2.3 ms, up to a value of 4.6 ms. These increases above that predicted for the monosynaptic branched-axon model may involve temporal dispersion in either or both common input fibres or peripheral nerves, or could also include an extra synapse. However, since we do not know the identity of the presynaptic axons involved, we cannot say how likely it is that an extra synapse is involved. However, note that it is not likely that a difference in preparation or state is likely to explain the difference between the values for T8/L1EO (represented in only two of the preparations) and those for T8/L1IO or T8/L1Dist, since this difference is evident within those two individual preparations (see values connected by lines in Fig. 6a).

#### Lags of histogram peaks

The lags were measured to the maximum values of the smoothed peaks and are summarized in Fig. 6b. For the ipsi-segmental thoracic nerve pairs, the lags were within 0.9 ms of zero. Those for the T8/T9 nerve pairs showed a positive bias (mean 0.36 ms), appropriate to an origin in descending common inputs. The lags for L1IO/L1Dist were scattered either side of zero, but those for L1EO/L1IO and L1EO/L1Dist showed a positive bias, consistent with the differences in nerve lengths and conduction times (Road et al. 2013). The relatively large positive lags for all of the T8/L1 nerve pairs, like those for the T8/T9 pairs, correspond to an origin in descending common inputs, with the four values for the T8EO/L1EO or T8Dist/L1EO being the lowest (1.7–2.1 ms), the one value for T8Fil/L1EO being only a little longer (2.5 ms) and with the rest ranging from 2.3 to 5.0 ms.

**Fig. 7:**
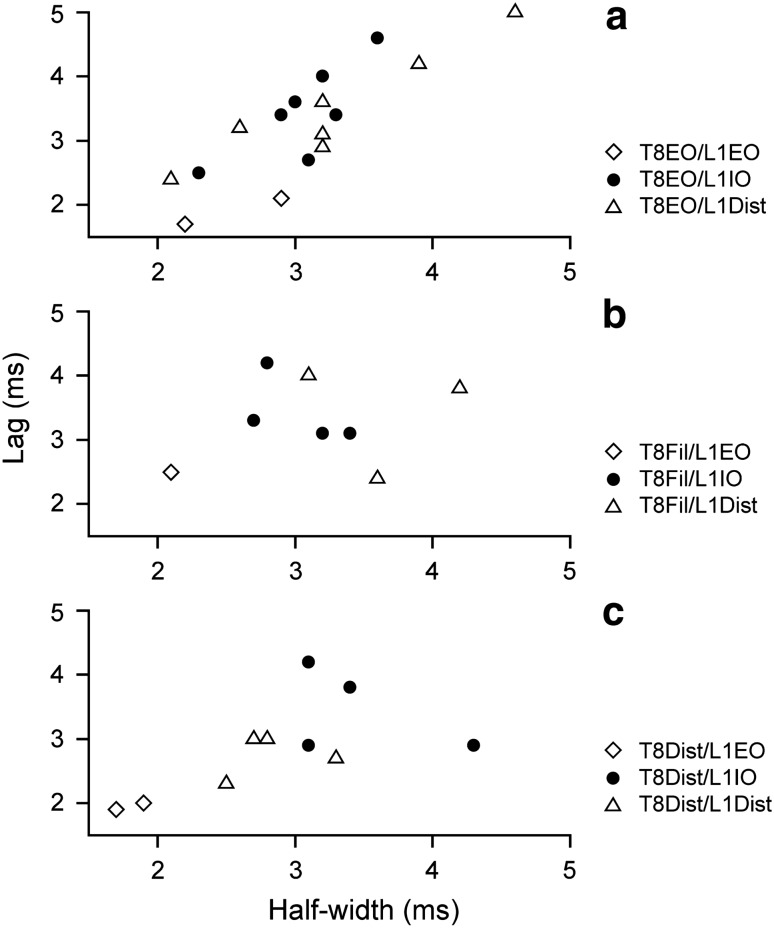
Relationships between lags and half-widths for T8/L1 histogram peaks. Different nerve pair categories, as indicated. Regression analysis showed a significant positive correlation for the T8EO/L1IO and T8EO/L1Dist categories

For any one category of nerve pair, there is a large scatter in the values of both half-width and peak lag in Fig. 6, particularly for the T8/L1 categories, but it was noticed that the values for these two parameters co-varied, at least for some categories, as illustrated in Fig. 7. This was particularly the case for those involving T8EO (a), where a significant positive correlation was present, both for T8EO/L1IO and for T8EO/L1Dist (*r*^2^ = 0.623 and 0.870, respectively; *P* = 0.035 and 0.002, respectively). The values for T8Dist/L1 nerve pairs (c) look like they might fit within a similar relationship, but, even when they were all are considered as one population, they gave a value for *r*^2^ of only 0.358, not quite significant (*P* = 0.068). No relationship was detected for the values from T8Fil/L1 (b). At least for the T8EO/L1 nerve pairs, it therefore seems likely that whatever factors created the longer lags also created the wider peaks.

### Rat experiments

#### Correlations between pairs of internal intercostal nerves

These are the correlations which are most readily compared with those in the cat described above. They were available in 10/11 of the preparations here. Eight of these were from nerves in adjacent segments, together with one example with T8/T10 and one with the T9Fil paired with T9Int.

**Fig. 8:**
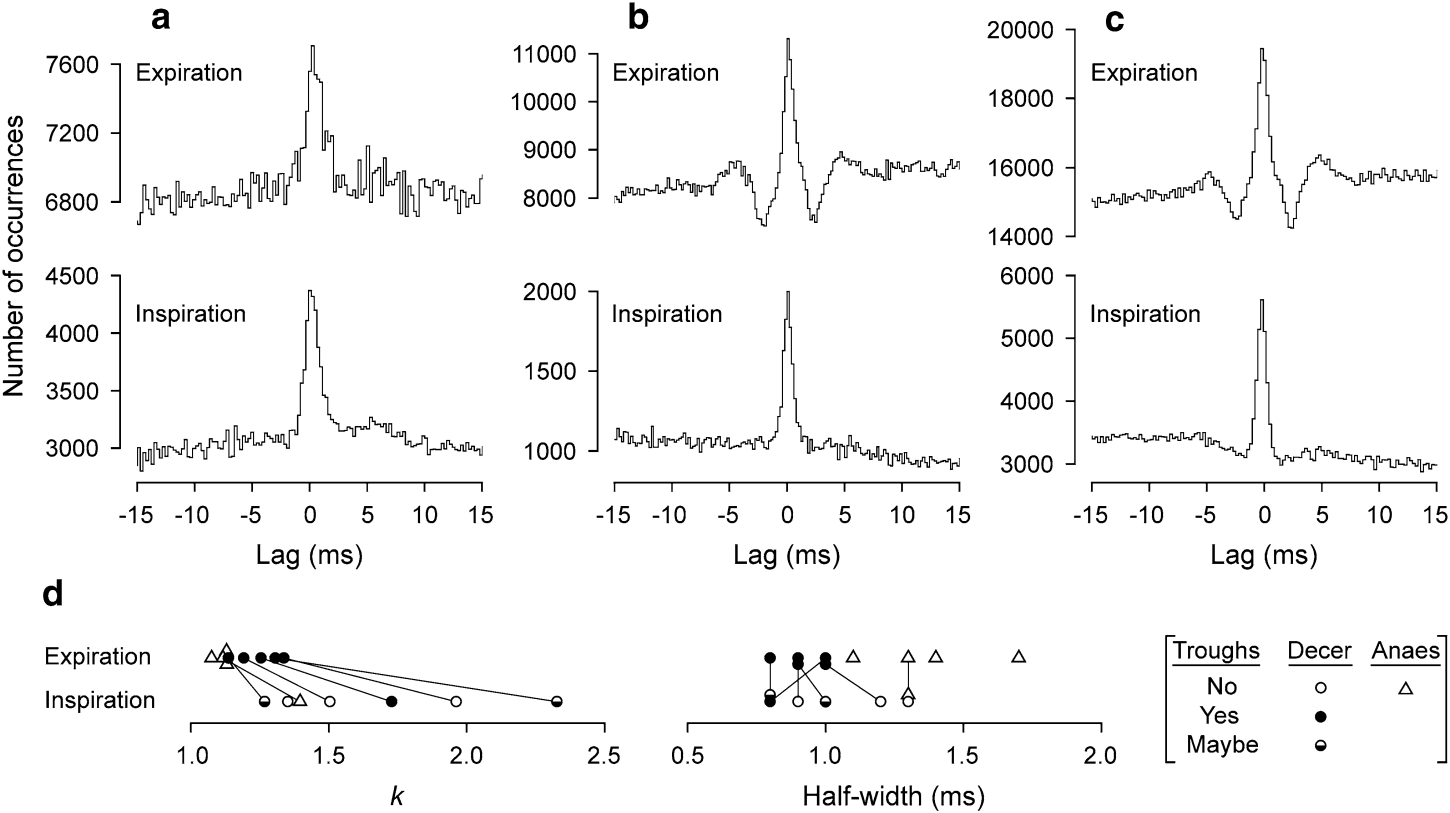
Examples of cross-correlation histograms from pairs of Int nerves in rat experiments. Pairs of histograms, each pair from a single recording, the upper histograms from expiration, the lower ones from inspiration. **a** Anaesthetized animal (T9Int/T10Int, data illustrated in Fig. 2). **b** Decerebrate (T9Int/T10Int), flanking troughs present in the histogram from expiration, but not from inspiration. **c** Decerebrate (T10Int/T11Int), flanking troughs for both. **d** Distributions of the values of *k* and half-width for all of the significant peaks from Int/Int nerve pairs. Lines join values from the same recordings in expiration and inspiration. The occurrence of flanking troughs indicated by symbols (“Maybe” means that the baseline noise in the histogram from inspiration could have hidden troughs of equivalent amplitude to those seen in the histogram for expiration)

As in the cat, narrow central peaks were present in the cross-correlation histograms of all of these (Fig. 8), mostly during both inspiration and expiration, though two anaesthetized animals were counted as not tested during inspiration (values of *m* < 121) and the peak for inspiration was not significant in one anaesthetized animal nor for expiration in one decerebrate (both relatively low *m*). The synchronization during expiration was of similar strength to that in the cat, *k* varying between 1.075 and 1.338, but included stronger examples during inspiration (up to 2.327). For the 6 instances where *k* was measured in both expiration and inspiration, it was always greater for inspiration (Fig. 8d). During expiration, *k* was greater in all of the 5 decerebrates than the 4 anaesthetized animals. Half-widths were generally shorter than in the cat, 0.8 to 1.7 ms, especially for the decerebrates (Fig. 8d). A notable feature, not seen in the cat and seen here only in the decerebrates, consisted of troughs on either side of the central peak, which we will refer to as “flanking troughs”. There were sometimes also weaker peaks on the other sides of these troughs, but never further (periodic) peaks or troughs. Flanking troughs were present for all of the histograms for pairs of Int nerves during expiration in decerebrates (e.g. Figure 8b, c) and were present in 1/6 of those during inspiration (Fig. 8c). The troughs for inspiration in Fig. 8c look weak in comparison with the central peak, but when measured with respect to *m* give similar *k* values to those during expiration, namely 0.938 and 0.931 in inspiration vs*.* 0.939 and 0.913 for expiration. In two other examples, because of relatively low values of *m* for the peaks assessed in inspiration, the noise in the histograms could have concealed troughs as large as those seen in the histograms from expiration (Fig. 8d, half-filled symbols). The remaining three histograms assessed in inspiration showed no signs of troughs (e.g., Fig. 8b). The lags to the maxima of the peaks varied from − 0.1 to + 0.2 ms, mean + 0.10 ms (*N* = 14) for nerve pairs in adjacent segments, 0 ms for the pair in the same segment and + 0.3 ms for the T8/T10 pair.

#### Correlations between external and internal intercostal nerves (caudal segments)

**Fig. 9:**
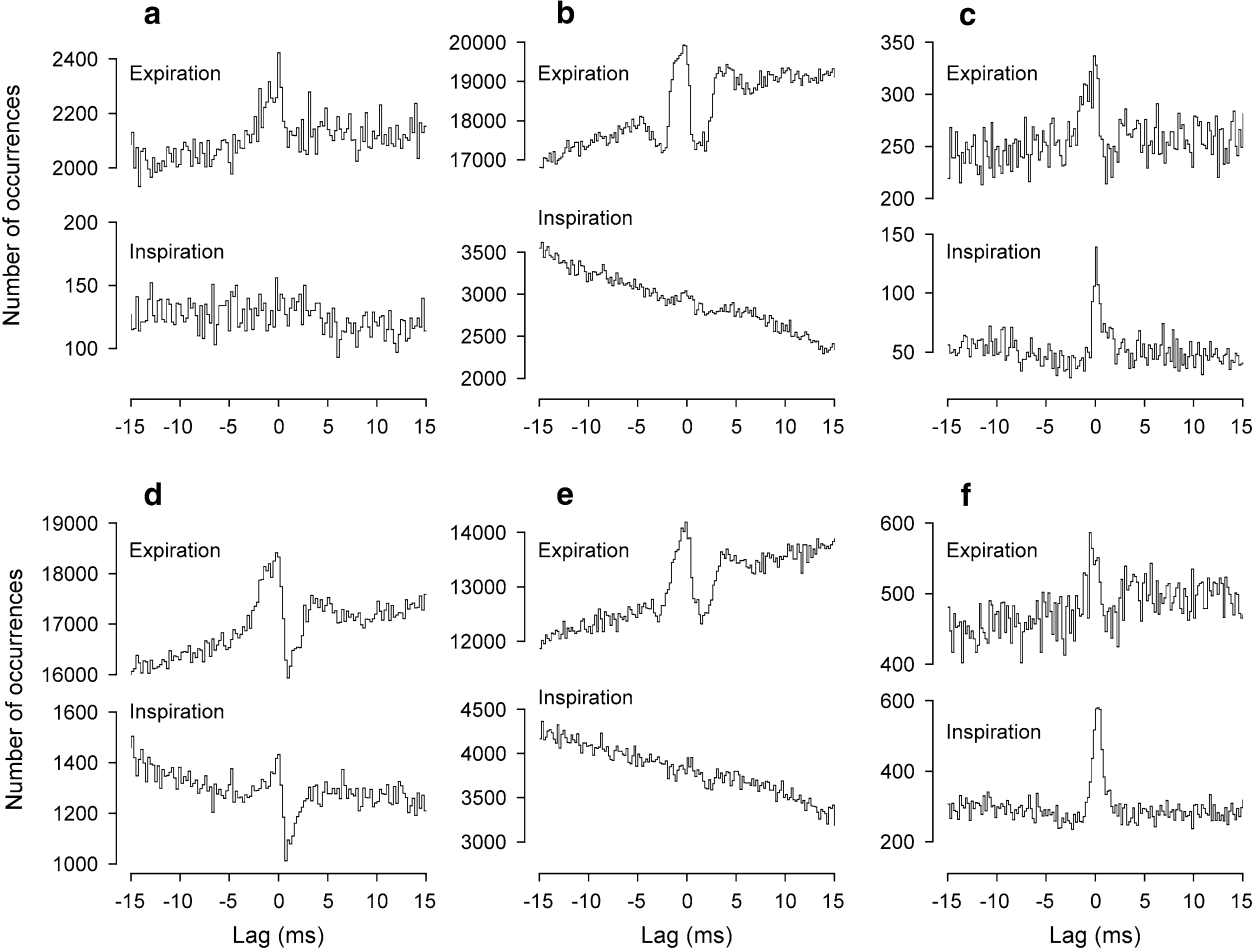
Examples of cross-correlation histograms from Ext/Int nerve pairs in rat experiments. Pairs of histograms, each from a single recording, the upper ones from expiration, the lower ones from inspiration. **a** Anaesthetized animal (T9Ext/T9Int, data illustrated in Fig. 2). **b**–**f** Decerebrate: **b**, **c** T9Ext/T9Int; **d** T10Ext/T10Int. **e** T9Ext/T10Int, same recording as (**b)**; **f** T9Ext/T10Int, same recording as (**c**)

These measurements was made for the Ext and the Int nerves in the same segment in 10 of the 11 animals (11 nerve pairs) and also from an equivalent pair in different segments in 9 animals (10 nerve pairs, 7 of which were from adjacent segments). For 10/11 of those in the same segment tested during expiration, a peak was observed, and in all instances, the peaks were asymmetric. In three of those in the anaesthetized animals and in one of the decerebrates, the asymmetry consisted of a maximum near zero lag, with a shallow fall to the left but a fast fall to the right (Fig. 9a, upper histogram), rather similar to that reported for correlations between inspiratory activities in external and internal intercostal nerves in the rostral segments in the cat (Vaughan and Kirkwood 1997). The reference events were always derived from the Ext nerve spikes. In the remainder (1 from an anaesthetized animal and 5 from decerebrates), the asymmetry consisted of a peak to the left, combined with a trough to the right (Fig. 9b–d, upper histograms). During inspiration, there was considerable variation in the features observed in the histograms, as illustrated in the lower histograms in Fig. 9a–d. Four nerve pairs were counted as not tested (*m* < 121), three gave featureless histograms, two showed weak peaks (one included in Fig. 9b), and the other two gave the histograms in Fig. 9c, d. One of these, strictly speaking, should have been counted as not tested (*m* = 50), but is illustrated in (c), because it showed the highest value of *k* observed in this study (2.78), and the other (d) showed a particularly sharp peak/trough combination.

A similar pattern was seen in the Ext/Int nerve combinations in different segments. Peaks were observed in the histograms from expiration from nine nerve pairs, which, individually, were remarkably similar to those for the histograms from same segment pairs in the same animals, where available, a similarity that was extended to also apply to the time courses seen during inspiration, as is clear in the examples in Fig. 8e (similar to b) and Fig. 8f (similar to c).

#### Correlations between T6 external nerve and nerves in the caudal segments

**Fig. 10:**
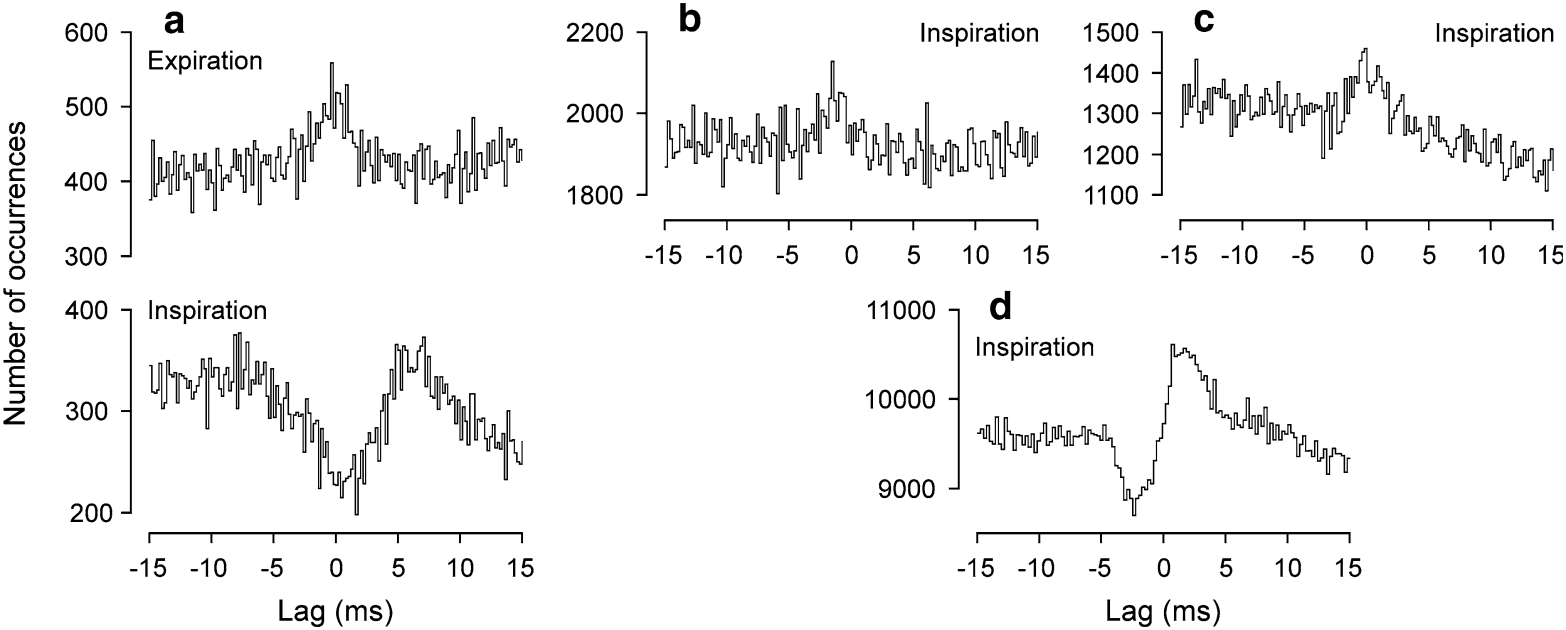
Examples of cross-correlation histograms with T6 Ext paired with other Ext nerves in rat experiments. **a** T6/T9: upper histogram, expiration; lower histogram inspiration. **b**–**d** inspiration only: **b** T6/T8; **c** T6/T9; **d** T6/T10. **b** anaesthetized; others, decerebrate

T6Ext was paired with a more caudal Ext nerve in 10 animals, T9 in 8, T8 in 1, and T10 in 1. Because expiratory activity in T6 was usually weak, only 2 pairs could be counted as tested during expiration and only 1 of these (from a decerebrate) gave a significant peak in expiration (Fig. 10a, upper histogram), but this is of interest because the histogram in inspiration showed a completely different time course (lower histogram), dominated by a rather broad trough. During inspiration, 9 were counted as tested and 8 showed significant features. Two of these were from anaesthetized animals, yielding histograms with central peaks (Fig. 10b). Four of the six from decerebrates gave similar peaks (Fig. 10c), but the other two gave trough/peak combinations, though at different lags (Fig. 10a, lower histogram, d). For the simple peaks during inspiration (*N* = 6), mean values of parameters were: *k*, 1.08 ± 0.04 (range 1.029–1.150); half-width, 1.75 ± 0.89 (range 1.2–3.0); lag to the maxima, − 0.68 ms ± 0.89 (range − 2.2 to + 0.1).

**Fig. 11:**
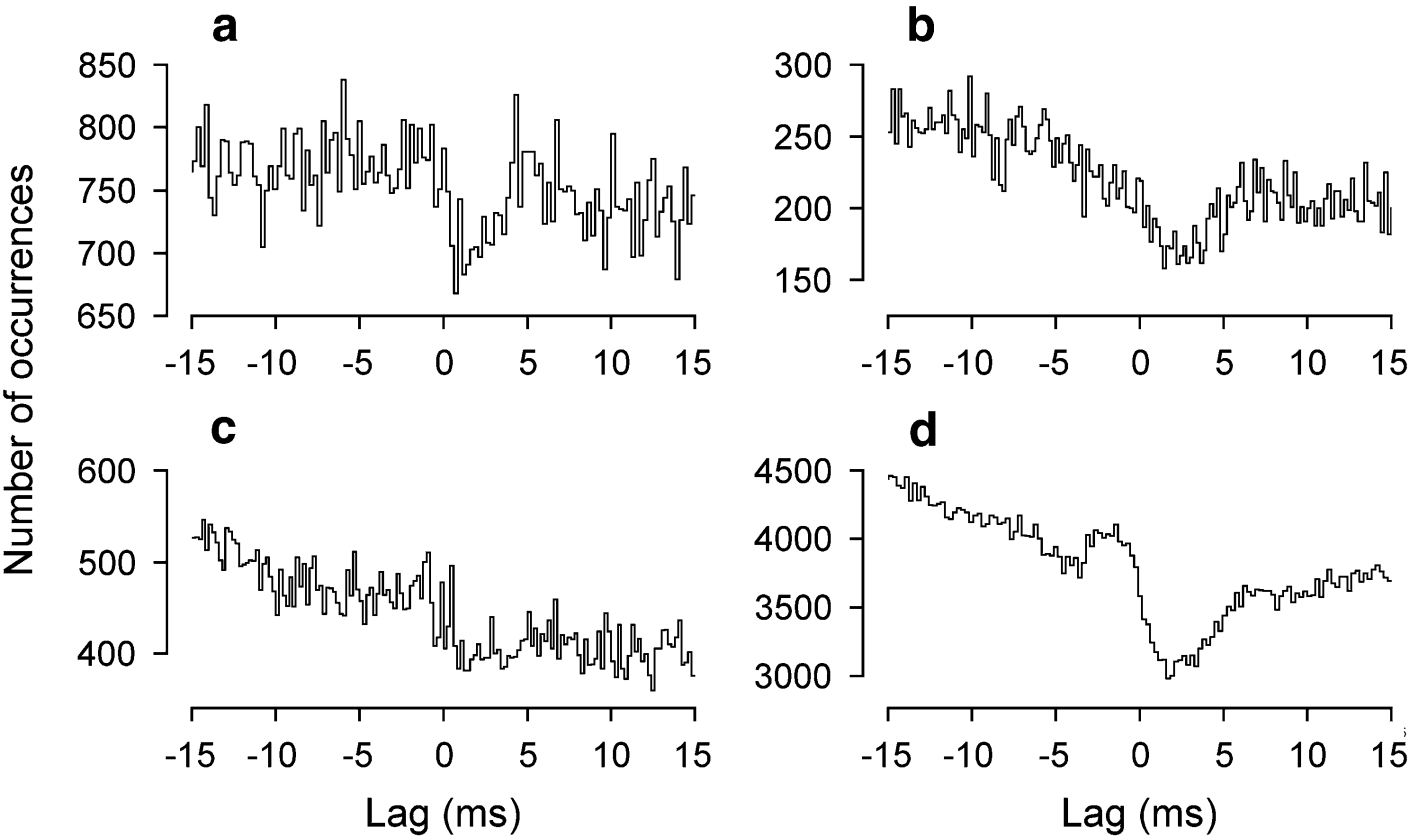
Examples of cross-correlation histograms with T6 Ext paired with caudal Int nerves in rat experiments. All measured in inspiration. **a** anaesthetized animal, T6Ext/T10Int (data illustrated in Fig. 2). **b**–**d** Decerebrates: **b** T6Ext/T9Int; **c**, **d** T6Ext/T10Int

T6Ext nerve was also paired with one or two of T8Int, T9Int, or T10Int nerves from the recordings from ten animals. Because of low counts in the histograms, only four examples were counted as tested during expiration. Two gave visible peaks just to the left of zero, but neither was significant. During inspiration, 13 pairs were tested (from 9 animals). Eight of these showed significant troughs to the right of zero (Fig. 11a, b), including 3 from anaesthetized animals, together with 2 others with clear troughs that were not significant. The remaining 3 histograms included two with peaks to the left of zero (Fig. 11c) and 1 with a peak/trough combination (Fig. 11d).

## Discussion

In this study, the expiratory motoneurones in the cat, which receive a strong monosynaptic descending expiratory drive, were sometimes found to show synchronization with narrow central peaks appropriate to monosynaptic branched-axon inputs, but this was not necessarily the dominant effect and plentiful examples of wider peaks, indicative of shared disynaptic or other connections were also found. On the other hand, for the expiratory motoneurones of the internal intercostal nerves in the rat, where the long descending monosynaptic connections are much weaker, rather strong synchronization showing only narrow peaks appropriate to monosynaptic branched-axon inputs was found. Other combinations of nerves in the rat gave short-term synchronization with more complex time courses, suggesting a variety of shared input patterns. Before more detailed interpretations are considered, it is necessary to restate the criteria used.

First, all of the synchronization described here can come under the heading of short-term synchronization (STS), originally coined by Sears and Stagg (1976) and interpreted by them in terms of the branched-axon monosynaptic explanation. However, even then they allowed that “the effects of non-random synchronization of discharges of the presynaptic neurones” could contribute minor effects to STS. Thus, our use of STS does not restrict us to a particular connection model, but simply, as in Kirkwood et al. (1982b), to synchronization within ± 5 ms, which empirically defines it as separate from broad-peak synchronization, which was sometimes, though not often seen here (Fig. 4). When broad-peak synchronization was seen, this fitted with the description in Kirkwood et al. (1982b) as occurring over ± 20 ms or more. The synchronization defined as STS here may include, as well as branched-axon monosynaptic effects, the results of shared disynaptic connections (Vaughan and Kirkwood 1997), or the peripheral temporal dispersion effects likely for the L1 nerves, or the various trough/peak combinations seen in the rat experiments.

Second, we have retained the criterion adopted by Vaughan and Kirkwood (1997), that only peaks with half-widths ≤ 2.1 ms (or for here 2.3 ms) should be considered as resulting from the branched-axon monosynaptic model. The justifications for this remain the same as set out in that paper, the simplest and strongest argument being that (given that neurones presynaptic to motoneurones are themselves likely to have common inputs) the narrowest systematically occurring set of cross-correlation histogram peaks should be taken to represent the simplest branched-axon form of common input. A possible confound for that argument could be recurrent excitation between motoneurones, which has recently been demonstrated to cross segmental boundaries in the mouse (Bhumbra and Beato 2018). However, such a strong effect has not yet been demonstrated in the cat and the rostrocaudal spread of the effect in the mouse is about 1 mm, whereas a spread of about 10 mm (the typical length of a cat thoracic segment) would be required to produce a significant intersegmental synchronization such as documented in Vaughan and Kirkwood (1997). The half-width criterion is in any case supported by the known time courses of single-fibre EPSPs in motoneurones (particularly their rise times), via their conversion to a time course of probability of firing (primary correlation kernel) and a branched-axon model (for refs, see Vaughan and Kirkwood 1997). Various models or simulations have been proposed over the years, either for these kernels or for motoneurone synchronization itself, but whether based on the empirical formulation suggested by Kirkwood and Sears (1978) or on more refined approaches (Poliakov et al. 1997) or on direct measurements (Gustafsson and McCrea 1984), any of those simulations that suggested wider peaks (e.g., Datta and Stephens 1990; Bremner et al. 1991) have involved common EPSPs (or equivalent depolarizing transients) with slower rise times than have been measured for the majority of known single-fibre EPSPs in motoneurones. Of course, the rise times of some individual EPSPs are longer, but it is the spread of rise times of the vast majority of EPSPs that counts in this. Measurements of the 10–90% rise times for the monosynaptic EPSPs from EBSNs under the same conditions as in the present experiments were appropriately short, with only 2/55 being > 1 ms (Saywell et al. 2007). It should be noted that temporal dispersion effects that might be considered likely to increase the durations of a correlation peak, such as those occurring in different branches of presynaptic axons or in motoneurone axons, or variable delays in motoneurone firing times at threshold (Fetz and Gustafsson 1983) are taken into account in the primary correlation kernel estimations most relevant here, estimated from multi-unit input/output cross-correlations (Kirkwood and Sears 1982; Davies et al. 1985a; Kirkwood 1995; Road et al. 2013).

All that being said, the branched-axon monosynaptic effect is a special case. It is to be expected to be encountered because of the known presence of branched axons presynaptic to motoneurones. However, because of *likely* (but hardly proven) common inputs also to those last-order neurones, more indirect common inputs should also be expected, leading to the inevitable broadening of the correlation peaks. Many studies in the literature include some peaks that would fit our criterion for a branched-axon monosynaptic effect, but also many that were wider (e.g., Datta and Stephens 1990; Bremner et al. 1991; Schmeid et al. 1993, 2000; Smith et al. 1999; Huesler et al. 2000; Hansen et al. 2001; Kilner et al. 2002; Keen et al. 2012; Westad and Westgaard 2005). We suggest that it is a matter of good fortune for our interpretations that the systematically occurring set of correlations in Vaughan and Kirkwood (1997) between rostral internal intercostal inspiratory (presumed interchrondral) motoneurones and between phrenic motoneurones indicated the branched-axon monosynaptic connections as a dominant effect in the conditions of those experiments. Even in that study, the effect was more mixed for caudal segments. Thus, mixed effects, as apparent for the cat data in the present study, represent the default condition. It must be emphasised that the 2.1 ms or 2.3 ms boundary for half-width should not be taken as absolute. It was empirically derived as the maximum observed for a particular population taken by Vaughan and Kirkwood (1997) as likely representing the branched-axon model, and thus, the maximum value safely to be considered as representing that model. Values below that may well also arise via a common disynaptic input and, moreover, the distinction should only be made on a population basis, rather than on an individual measurement. It is the distribution of half-widths that counts. Those readers that may think that the effects of common di- or oligosynaptic connections are too weak to appear in motoneurone synchronization and who therefore hesitate to accept the arguments put forward by Vaughan and Kirkwood (1997), should be convinced by the complex waveforms seen in the rat data here, which presumably involve inhibition, and which are very hard to explain without oligosynaptic linkages. We suggest that the positive evidence for oligosynaptic connections derived from the synchronization time courses easily outweighs lingering doubts about likely weaknesses of effects, for which there is little direct evidence.

For motoneurone synchronization in general, a number of authors have cautioned against concluding that a difference in its strength represents a difference in the proportion of common inputs, on account of the many factors that contribute to the form of the primary correlation kernels in motoneurones, such as variations in the amplitude and frequency content of the synaptic noise, the motoneurone threshold, modulation via activation of intrinsic membrane channels, or the motoneurone firing rate (Ellaway and Murthy 1985; Nordstrom et al. 1992; Poliakov et al. 1996; Binder and Powers 2001; Taylor and Enoka 2004; Rodriguez-Falces et al. 2017). These factors no doubt will also influence last-order interneurones, thus further adding variation to the strength of synchronization via oligosynaptic common inputs. In the discussion that follows, we will therefore make only limited comparisons between strengths of correlation, relying mostly on the time courses of the peaks or troughs.

### Cat experiments

Considering the thoracic nerve pairs, the main result here is that, although many of the measurements showed effects appropriate to branched-axon monosynaptic common inputs (i.e., peaks with half-widths below 2.3 ms), a substantial proportion did not. The EBSNs of caudal nucleus retroambiguus are generally regarded as the only group of neurones transmitting the excitatory expiratory drive to the spinal cord and are known to monosynaptically excite the motoneurones. Thus there are two processes that could lead to the longer duration STS peaks, either synchronization of these EBSNs or synchronization of other inputs to the motoneurones. With respect to the former, we have the advantage that in 9/12 of the cat experiments, connections between EBSNs and motoneurones were also measured and synchronization between the EBSNs, as reflected in “medium-width” peaks in the EBSN/motoneurone cross-correlations, was seen to be infrequent (Road et al. 2013; also see Kirkwood 1995). We therefore favour the latter explanation.

One possibility is that of disynaptic connections from the EBSNs to the motoneurones, where the EBSNs are thus the synchronizing influence on presumed last-order interneurones. The mixture of narrow (half-width ≤ 2.3 ms) plus wider components in the motoneurone synchronization would then reflect a mixture of mono- plus disynaptic connections from the EBSNs. However, a second possibility comes from the calculations made by Saywell et al. (2007), which suggested that a non-respiratory input was necessary in addition to the phasic expiratory drive to bring the expiratory motoneurones to threshold. Both of these possibilities suggest a dependence on spinal interneurones, many of which are active in preparations such as used here (Kirkwood et al. 1988).

Considering the L1 nerve recordings, the synchronization measurements here are fully consistent with the views above. The long durations for many of the histogram peaks were much too long to be explained by branched-axon monosynaptic common inputs, even allowing for peripheral temporal dispersion. The long lags apparent for the peaks from the T8/L1 nerve pairs (Fig. 6b) are also consistent with oligosynaptic linkages. These suggest a descending drive, but the lags were larger than could be accounted for by the conduction velocities in the EBSNs, which would be likely to account for only about 2 ms (Road et al. 2013, their Fig. 2). The covariation between half-width and lag in Fig. 7 is also consistent with an oligosynaptic linkage, of variable extent.

One detail in this variation in the lumbar nerve measurements is of interest. This is that 4/5 of the T8/L1EO nerve pairs gave peaks with half-widths ≤ 2.2 ms and 4/5 with lags ≤ 2.1 ms, suggesting that the L1EO discharges were less dependent on our hypothesised interneuronal network. If the L1 EO motoneurones therefore received less excitation from such a network, then this would be consistent with the general low level of expiratory alpha motoneurone discharges in these nerves as compared to the other L1 nerves (Road et al. 2013), and would be a particular example of activity in the interneuronal network being responsible for the known differences in expiratory activity across the different regions of the thorax or abdomen, as proposed by Saywell et al. (2007).

### Rat experiments

In contrast to the cat, the synchronization shown by pairs of Int nerves showed exclusively narrow peaks (the longest half-width being 1.7 ms). During expiration, the synchronization was generally of similar strength to those in the cat (compare Fig. 8d with Fig. 5), but was noticeably stronger during inspiration. Note that, although we know that some individual motoneurones of the Int nerves are excited in both phases of respiration (de Almeida et al. 2010; de Almeida and Kirkwood 2010), we do not know how frequent that is. Thus, the same motoneurones may or may not contribute to the synchronization in both phases. However, the difference in the synchronization does suggest a different organization of the motoneurone inputs in the two phases, consistent with the appearance of separate waves of excitation with a clear transition between them in intracellular recordings (de Almeida and Kirkwood 2010). The different organization may be a difference in the common inputs, but also might be a difference in the overall synaptic noise (as observed by de Almeida and Kirkwood 2010), which could influence the translation of EPSPs to correlation kernels. Because the motoneurones of these nerves show a strong expiratory drive, but receive only weak EBSN monosynaptic inputs, we should assign the synchronization during expiration largely to spinal interneurone inputs. For these motoneurones during inspiration, there is no direct evidence for the origin of their excitation, though since inspiratory bulbospinal motoneurones in the rat do not give direct excitation to Ext motoneurones (Tian and Duffin 1996), a spinal interneurone input again seems the most likely.

An interesting feature of the synchronization for pairs of Int nerves was the presence of flanking troughs, though only in the decerebrates. Examples similar to these in motoneurone synchronization for various categories of motoneurones can be found in individual illustrations scattered through the literature (Bremner et al. 1991; Tian and Duffin 1996, their Fig. 4; Vaughan and Kirkwood 1997, their Fig. 2; Peever et al. 2001, their Fig. 2; Root and Stephens 2003; Sandhu et al. 2009, their Fig. 2), though this time course has been hardly discussed. Like the examples in the present experiments, the flanking troughs in these examples were all narrow, with a rapid onset and offset, unlike those reported by Farmer et al. (1993, their Fig. 2B) from a stroke patient, with a very narrow peak and slow “offset” to the troughs. This time course can be explained by underlying primary correlation kernels with negative overshoot (e.g. Fetz and Gustafsson 1983; cf. Türker and Powers 2002), likely arising from particular combinations of common EPSPs and synaptic noise. On the other hand, we have included Bremner et al. (1991) in the above list as being similar to the present examples because of the appearance of some of the illustrations in that report and because it is the one instance where the time course was actually discussed. However, the description in the text of that paper refers to rather longer duration troughs and the explanation the authors offer for troughs with a longer duration is a very reasonable one, that of a mapping of the autocorrelation of the discharges of the individual motoneurones involved. This could not fit the present examples. However, one possible explanation that could be offered for these is that of an effect from the autocorrelations of the discharges of the common input neurones, so long as these were sufficiently fast firing, with modal interspike intervals of the order of 5 ms or less. This is an attractive hypothesis because the difference between the anaesthetized and decerebrate examples might then correspond to the big difference in firing frequencies seen between the EBSNs in these two particular preparations (de Almeida et al. 2013). The assumption here is that the interneurones assumed to be providing most of the common inputs showed a behaviour similar to the EBSNs, which is a reasonable assumption given descriptions of other respiratory neurones in decerebrate rats. These descriptions also include very fast firing rates (e.g., Duffin and van Alphen 1995).

However, a second possibility is that inhibition is involved in the troughs. Indeed, the time course here is remarkably similar to that of the synchronization between pairs of Bötzinger complex expiratory neurones (Tian et al. 1999), where the interpretation was of mutual inhibition. This was a reasonable interpretation, given that most of these neurones are known to be inhibitory and that, in some instances, the troughs were seen without the central peak. In the present data, of course, the presumed mutual inhibition could not be direct, but could be recurrent, via Renshaw cells, even though the time course here is rather different from the various examples in the modelling study of Uchiyama and Windhorst (2007). Alternatively, inhibition could be part of a feed-forward circuit, such as excitation of one motoneurone group being common with disynaptic inhibition of the other. Without more information, it is hard to decide between the input autocorrelation and the inhibition explanations, but the inhibition explanation is particularly attractive because of the observations of troughs for the other categories of motoneurones, for which the interpretation of inhibition is hard to avoid (see below).

The second group of correlations investigated are the Ext/Int nerve pairs of the same or adjacent segments (Fig. 9). The asymmetric peaks seen here during expiration can readily be interpreted, as in Vaughan and Kirkwood (1997), as resulting from common inputs that are direct to the motoneurones of the Int nerve but disynaptic to those of the Ext nerve. This would be consistent with the connections from EBSNs seen by de Almeida and Kirkwood 2013) as direct to Int motoneurones but disynaptic to Ext motoneurones. Given the relative weakness of these EBSN connections, an assumption that is then necessary is that the interneurone connections presumed now to be mostly responsible for the synchronization should follow the same pattern. The troughs present on the right side of some of these histograms could be explained as representing expiratory inhibition of the Int motoneurones in parallel with their excitation. If so, then these observations would confirm the few intracellular observations of parallel excitation and inhibition during expiration in these motoneurones reported by de Almeida and Kirkwood (2010). The more frequent observations of the same phenomenon during inspiration in that same study seem to be hardly represented in the synchronization for these nerve pairs during inspiration, except, perhaps, for Fig. 9d (though see below for pairings with T6Ext).

Note that strict logic does not allow us to say with certainty that the presence of a trough implies inhibition nor, even if so, to say for sure which of the two motoneurone groups was inhibited. However, with troughs mostly on the right side of the histograms and the reference spikes being from the Ext motoneurones, the simplest explanation is to suggest common monosynaptic excitation of the Ext motoneurones with disynaptic inhibition of the Int motoneurones. One might consider adding an extra synapse to either branch of such a circuit, but not more, given the sharp and relatively short durations of several of the troughs illustrated in Fig. 9. The inhibition could be either feed-forward or recurrent, perhaps mutual for Fig. 9b. The variable patterns of excitation and inhibition seen in the histograms are probably related to the variability of patterns seen in the nerve discharges (de Almeida et al. 2010), but it is of interest that within individual preparations, consistency was seen between the pairs of nerves from different segments, including both in expiration and inspiration (Fig. 9).

The final group of synchronization measurements includes those involving T6Ext paired with the more caudal Ext or Int nerves, nearly all made during inspiration. Those with the Ext nerves (Fig. 10) mostly showed peaks, some short enough to correspond to branched-axon common inputs, others with longer duration, i.e., similar overall to those in the cat (Kirkwood et al. 1982a), or to the Int nerves here. However, an important difference was that there was not a positive lag to the histogram peaks that would correspond to a common input from descending fibres. Tian and Duffin (1996) made a similar observation for more rostral segments. Note that such a lag should be readily detectable (Kirkwood et al. 1982a). Once more, this puts the emphasis on local interneurones as the source of the synchronization, again consistent with the absence of direct inspiratory bulbospinal inputs reported by Tian and Duffin (1996).

Two of the histograms in the present data included clear troughs (Fig. 10a, d). We have no specific interpretation for these, but either of the previously mentioned possibilities of inhibition, recurrent or feed-forward, could apply.

For the T6Ext pairings with the Int nerves, the dominance of troughs to the right of zero can be taken as evidence for inhibition of the Int motoneurones during inspiration, as in the cat (Sears 1964b) and confirming the parallel excitation and inhibition reported by de Almeida and Kirkwood (2010).

Note that the interpretations offered above in terms the circuits involved are clearly provisional. We have not attempted detailed modelling such as in Vaughan and Kirkwood (1997), it being almost certain that a wide variety of different models could be fitted. However, one further point must be added, given the presence of widespread inhibition, that a contribution to any of the *peaks* from common inhibition (generally mathematically equivalent to common excitation) cannot be ruled out.

### Functional considerations

The spinal interneurone effects on synchronization described here imply the presence of active interneurone inputs to the motoneurones in both the cat and rat preparations, and thus support the roles of interneurons that have been previously proposed to be important in the generation of the spatial and temporal patterns of intercostal and abdominal motor activity, both in humans (Hudson et al. 2011) and animals (Davies et al. 1985b; Tian and Duffin 1996; Saywell et al. 2007; de Almeida and Kirkwood 2013). However, the strengths of synchronization here were relatively weak, thus supporting the view of vertebrate spinal cord interneurone networks as being sparse (Nielsen et al. 2005; Radosevic et al. 2019), itself consistent with the projection patterns of individual thoracic interneurons (Saywell et al. 2011). The concurrent inhibition and excitation in the rat motoneurones here might be interpreted as an example of balanced excitation and inhibition, which has been proposed as a general principle in the organization of motor systems (Berg et al. 2019). Our observations of peak/trough combinations could support this idea, so long as the peaks and troughs were really linked, i.e., if the same common inputs were responsible for both. However, we have no evidence for whether or not that is the case.

The widespread appearance of troughs in the histograms from the rats represents a clear difference in comparison with those from the cats. Does this represent a species difference or a state difference, such as anaesthesia vs. decerebration? Activities in interneurone networks are well recognized as being under descending or modulatory control and state dependence might be suspected because most of the troughs came from the decerebrate preparations. However, there were also some examples of troughs from the anaesthetized animals (e.g., Fig. 11a) and, in any case, rather few anaesthetized animals were tested. Moreover, it is probably not the inhibition that is absent in anaesthetized animals compared with the decerebrates, since both inspiratory and expiratory thoracic motoneurones in anaesthetized cats are known to be inhibited during their “off” phases of the respiratory cycle (Sears 1964b). Thus, the major difference in the rat may be an additional phase of excitation superimposed on this, allowing the motoneurones to fire in both phases of respiration. In the rat, such excitation is present in both the anaesthetized and decerebrate states (de Almeida and Kirkwood 2010). Event in the decerebrate cat, such diphasic excitation has not been observed in thoracic recordings (Cohen et al. 1985; Enríquez Denton et al. 2012), nor have any troughs been reported in the few measurements of motoneurone synchronization made from expiratory intercostal discharges in cats under similar conditions (Sears and Stagg 1976; Kirkwood et al. 1982b).

We therefore suggest that the differences reported here reflect real connectivity differences between the species. A great deal of new information has emerged in recent years from experiments in the rodents, including new definitions of interneurone classes defined by factors such as their developmental lineages. These different classes will have clear homologies across species. However, the present experiments add to the warnings already expressed (de Almeida and Kirkwood 2013; Radosevic et al. 2019), that major species differences in the connectivities of those interneurons may well be present and may well, therefore, have implications for translational studies, such as in experimental spinal cord repair.

### Broader implications

#### Human *vs* animal studies

The most widespread use of motoneurone synchronization measurements is in human studies, as a way of accessing information on the last-order connections to motoneurones. For instance, Nielsen (2016) cited STS, interpreted via the monosynaptic branched-axon model, as part of the evidence supporting the predominance of corticomotoneuronal connections in voluntary movements. We have already discussed the relevance of the present results in qualifying the monosynaptic branched-axon interpretation, but it is worth pointing out the particular advantage of the animal recordings in this respect. In particular, the intercostal nerve recordings involve short conduction distances, which contribute little peripheral temporal dispersion, so multi-unit recordings can be used without distorting the time courses of the synchronization, thus making them comparable to the typical paired single-unit analyses employed in human recordings. This factor, combined with long recording runs, allows high bin counts with narrow bin-widths, while maintaining a respectable signal-to-noise ratio in the cross-correlation histograms. The sub-millisecond resolution thus achieved is an important part of the critical distinctions that we are able to draw. These animal recordings thus form an important link between invasive single-unit connectivity measurements in animals and the indirect measurements in humans.

#### Inhibition

As far as we know, the troughs here are almost completely new observations for motoneurone synchronization measurements. The only comparable observations come from the relatively narrow troughs seen for co-contracting human soleus and tibialis anterior muscles in the Stephens laboratory (Gibbs et al. 1994a, b), interpreted as arising from the well-known reciprocal inhibitory connections for these antagonistic muscles. However, these troughs are not always seen. Publications from the Nielsen laboratory (Neilsen and Kagamihara 1994; Hansen et al. 2002) reported few troughs (and these of relatively long duration). More often peaks, of various durations, were present. The difference may have arisen because the subjects in the Stephens lab were mostly standing, with toes raised (Mayston et al. 1996), whereas those in the Nielsen lab were seated and performed voluntary co-contractions with feedback of their torque and EMG levels, for which the configurations of the interneurone circuits were likely to have been more strictly controlled from the cortex.

Inhibition is a vital part of motor control, so it is a pity that methods such as motoneurone synchronization measurements have to date yielded so little information in this respect. The present results show that in principle they can. The actions that are studied will need to be those where, as here and as was presumably the case for Gibbs et al. (1994a, b), the necessary co-activation does not involve suppression of the inhibitory pathways.

#### Interpretations of peaks and troughs in cross-correlation histograms

Distinguishing direct synaptic effects from common input effects in cross-correlation histograms is a well-recognized problem. Davies et al. (1985a) pointed out that, in their measurements, they could distinguish between direct synaptic effects and those arising from presynaptic synchronization (i.e., common inputs) only because of the presence of a long conduction distance between the pairs of neurones concerned. They pointed out that without such conditions applying, deductions about connections that are made on the basis only of timing or durations of peaks or troughs in cross-correlation histograms are frequently unsafe. The observations of troughs here in motoneurone synchronization measurements (particularly those with relatively sharp troughs offset from zero) are uniquely valuable in this regard, since they cannot arise via direct connections.

A couple of examples are needed to make this clear. One has already been mentioned (Tian et al. 1999): compare their illustrations with our Fig. 8b, c. Another comes from a related publication: compare Fig. 4A, B from Peever et al. (2002) with our Fig. 9b–f. In both of these examples, the authors interpreted offset troughs remarkably similar to those in our recordings as indicating direct inhibition. Since this cannot be the case for the histograms here, then the similarity between the time courses in these two examples provide direct confirmation of what Davis et al. (1985a) argued a priori, i.e., that the deductions of the presence of direct connections from such waveforms are unsafe. There is no doubt that many other examples can be found in the literature of connections deduced via cross-correlation histograms from pairs of neurones in the CNS that, based on this reasoning, should be considered as fundamentally ambiguous. Interestingly, Tian and Duffin (1997), when reporting (from the same laboratory) very similar asymmetric waveforms to those in Peever et al. (2002), took a view similar to ours, that such waveforms were “difficult to interpret unambiguously”.

## Abbreviations

EBSN: Expiratory bulbospinal neurone
Ext: External intercostal nerve
Int: Internal intercostal nerve
T8Ext: External intercostal nerve of T8
T8Int: Internal intercostal nerve of T8
T8EO: Internal intercostal nerve branch at T8 that innervates external abdominal oblique muscle
T8Dist: Remainder of internal intercostal nerve of T8
T8Fil: Individual branch of internal intercostal nerve innervating internal intercostal muscle of T8
L1EO: Branch of L1 ventral ramus innervating external abdominal oblique muscle
L1IO: Branch of L1 ventral ramus innervating internal abdominal oblique muscle
L1Dist: A distal branch of L1 ventral ramus (see "Methods" section)
STS: Short-term synchronization
